# Unraveling the Carcinogenic Mechanisms of Food Contaminants: An Integrated in Silico Framework Combining Network Toxicology, Machine Learning, and Molecular Docking

**DOI:** 10.1111/1750-3841.70697

**Published:** 2025-11-17

**Authors:** Bangsheng Chen, Maomao Li, Yi Gu, Wenzhu Lou, Shuaishuai Huang, Feiyan Mao, Lian Tan, Zhiyan Wang

**Affiliations:** ^1^ Emergency Medical Center Ningbo Yinzhou No. 2 Hospital Ningbo Zhejiang China; ^2^ Urology surgery Ningbo Yinzhou No. 2 Hospital Ningbo Zhejiang China; ^3^ Ningbo Institute of Innovation for Combined Medicine and Engineering The Affiliated Lihuili Hospital of Ningbo University Zhejiang China; ^4^ Department of General Practice Ningbo Yinzhou No. 2 Hospital Ningbo Zhejiang China; ^5^ Laboratory of Renal Carcinoma Ningbo Yinzhou No. 2 Hospital Ningbo Zhejiang China; ^6^ Department of General Surgery Ningbo No. 2 Hospital Ningbo Zhejiang China; ^7^ Intensive Care Unit Ningbo Yinzhou No. 2 Hospital Ningbo Zhejiang China; ^8^ Department of General Surgery Ningbo Yinzhou No. 2 Hospital Ningbo Zhejiang China

**Keywords:** cancer, food contamination, machine learning, molecular docking, network toxicology

## Abstract

Food contamination poses a significant global health threat with carcinogenic potential, though the molecular pathways connecting contaminants to cancer remain poorly understood. This study sought to identify key molecular targets mediating the carcinogenic effects of nine prevalent dietary contaminants: glyphosate, perfluorooctane sulfonate, nitrosamines, pentabromodiphenyl ethers, methylmercury, dioxins, acrylamide, pyrrolizidine alkaloids, and aflatoxin. Using multiple online databases, we identified target genes associated with these contaminants and pan‐cancer, then conducted protein–protein interaction (PPI) analysis and visualization on intersecting genes. Subsequent gene ontology (GO) and Kyoto encyclopedia of genes and genomes (KEGG) functional enrichment analyses were performed to uncover potential mechanisms, with a focus on breast (BRCA), prostate (PRAD), and colon (COAD) carcinomas due to their significant pathway associations. Hub genes were prioritized through an integrative strategy combining topological algorithms in cytoscape (Centiscape, MCODE, and cytohubba's MCC), machine learning validation, and weighted gene co‐expression network analysis (WGCNA). Molecular docking simulations were conducted to examine interactions between contaminants and hub genes. The study identified 69 pan‐cancer‐intersected targets, with enrichment analyses revealing significant cancer‐associated pathways. Hub gene prioritization pinpointed JUN in BRCA, CDC42 in COAD, and MAPK14 in PRAD as critical regulatory targets. Validation using The Cancer Genome Atlas (TCGA) data confirmed statistically significant differential expression patterns (*p* < 0.05) for these targets across respective malignancies. Gene set enrichment analysis (GSEA) outlined pathway activation profiles consistent with tumor progression mechanisms. Molecular docking simulations demonstrated strong binding affinities (binding energy ≤ −5.0 kcal/mol) between contaminants and structural domains of the identified hub targets, suggesting potential mechanistic links between these food contaminants and cancer development.

**Abbreviations**
AFT: Aflatoxin
AM: Acrylamide
BRCA: Breast cancer
COAD: Colorectal cancer
EPA: Environmental Protection Agency
Gly: Glyphosate
GO: Gene ontology
GSEA: Gene set enrichment analysis
KEGG: Kyoto Encyclopedia of Genes and Genomes
MCC: Maximal clustering coefficient
MCODE: Molecular complex detection
MeHg: Methylmercury
NIEHS: National Institute of Environmental Health Sciences
NIT: Nitrosamines
PA: Pyrrolizidine alkaloids
PBDEs: Pentabromodiphenyl ethers
PCDDs: Dioxins
PFOS: Perfluorooctane sulfonate
PPI: protein–protein interaction
PRAD: Prostate cancer
TCGA: The Cancer Genome Atlas
WGCNA: Weighted gene co‐expression network analysis

## 1 Introduction

Cancer continues to be one of the most significant global health challenges, responsible for close to 10 million deaths each year, and current estimates indicate this number could rise by half by 2050 (Bray et al. 2024). While research in oncology has made substantial progress in uncovering the genetic and molecular mechanisms behind cancer development, studies show that 20–30% of cancer cases are associated with avoidable environmental risks like chemical exposures (Inamura et al. 2022; Kayamba and Kelly 2022). Despite growing recognition of this issue, many people remain unaware of common environmental carcinogens, such as air pollution, industrial chemicals, and contaminants in food, which continue to pose serious health threats. This lack of awareness leads to ongoing, preventable exposures that fuel the increasing cancer rates, underscoring the critical importance of better identifying and reducing these risks to protect public health.

Food contaminants represent a significant but frequently overlooked category of environmental carcinogens (Maher and Nowak 2022; Sadighara et al. 2024), with substances like glyphosate (herbicide residues), perfluorooctane sulfonate (PFOS, a persistent organic pollutant), nitrosamines (found in processed meats), and aflatoxins (produced by mold in improperly stored crops) being widespread in global food supplies (Bonato et al. 2025; Masci et al. 2024; Sipos et al. 2021; Vignesh et al. 2024). Research has clearly shown these compounds increase cancer risk, aflatoxin B1, for example, promotes liver cancer by disrupting cell death processes, while PFOS exposure is tied to higher rates of thyroid and lung cancers. Even common cooking practices pose risks, as acrylamide forms during high‐temperature food preparation and is recognized as a probable human carcinogen due to its genotoxic properties (Sassano et al. 2025; L. Zeng et al. 2025).

Traditional toxicology often struggles to unravel the complex, multi‐target mechanisms of foodborne carcinogens, but network toxicology overcomes this challenge by combining systems biology and computational modeling to map intricate chemical‐gene‐disease interactions and pinpoint central hubs in carcinogenic pathways (Cheng et al. 2025). For instance, Li et al. (2025) leveraged protein‐protein interaction (PPI) networks and machine learning to uncover critical genes, such as HDAC6, CDK1, DNMT1, NOS3, and DPP4, that mediate the impact of air pollutants on prostate cancer (Y. Li et al. 2025). Similarly, He et al. (2024) used network toxicology, protein interaction analysis, and molecular docking to clarify how plasticizers interact with key proteins involved in breast cancer, effectively tackling the “needle‐in‐a‐haystack” problem of target prioritization (He et al. 2024). Additionally, Xu et al. (2024) integrated differential expression analysis, weighted gene co‐expression network analysis (WGCNA), database mining, machine learning, and molecular docking to reveal the therapeutic mechanisms of Hypericum perforatum in major depressive disorder (MDD) (Xu et al. 2024). Their comprehensive approach identified crucial targets and pathways, particularly those related to immune function, offering a powerful framework for translating high‐dimensional data into actionable insights for disease prevention. Together, these methodologies provide a vital link between chemical exposure and disease outcomes, advancing our understanding of complex biological interactions.

Molecular docking simulations provide atomic‐level insights into carcinogen‐target interactions, effectively complementing network‐based predictions (Agu et al. 2023). For instance, Manal A. Abbas et al. (2024) revealed a high‐affinity binding interaction between bisphenol A and estrogen receptor α, offering valuable mechanistic clues about its involvement in breast cancer progression (Abbas et al. 2024). Similarly, Babak Arjmand et al. (2022) successfully applied molecular docking techniques to optimize ligand conformations, prioritize bioactive compounds, and identify promising anticancer candidates (Arjmand et al. 2022). The integration of docking results with network topology analysis enables researchers to verify key molecular targets and enhance mechanistic understanding, ultimately strengthening the framework for carcinogenic risk evaluation. This combined approach serves as a powerful methodological strategy in toxicological research.

**FIGURE 1:**
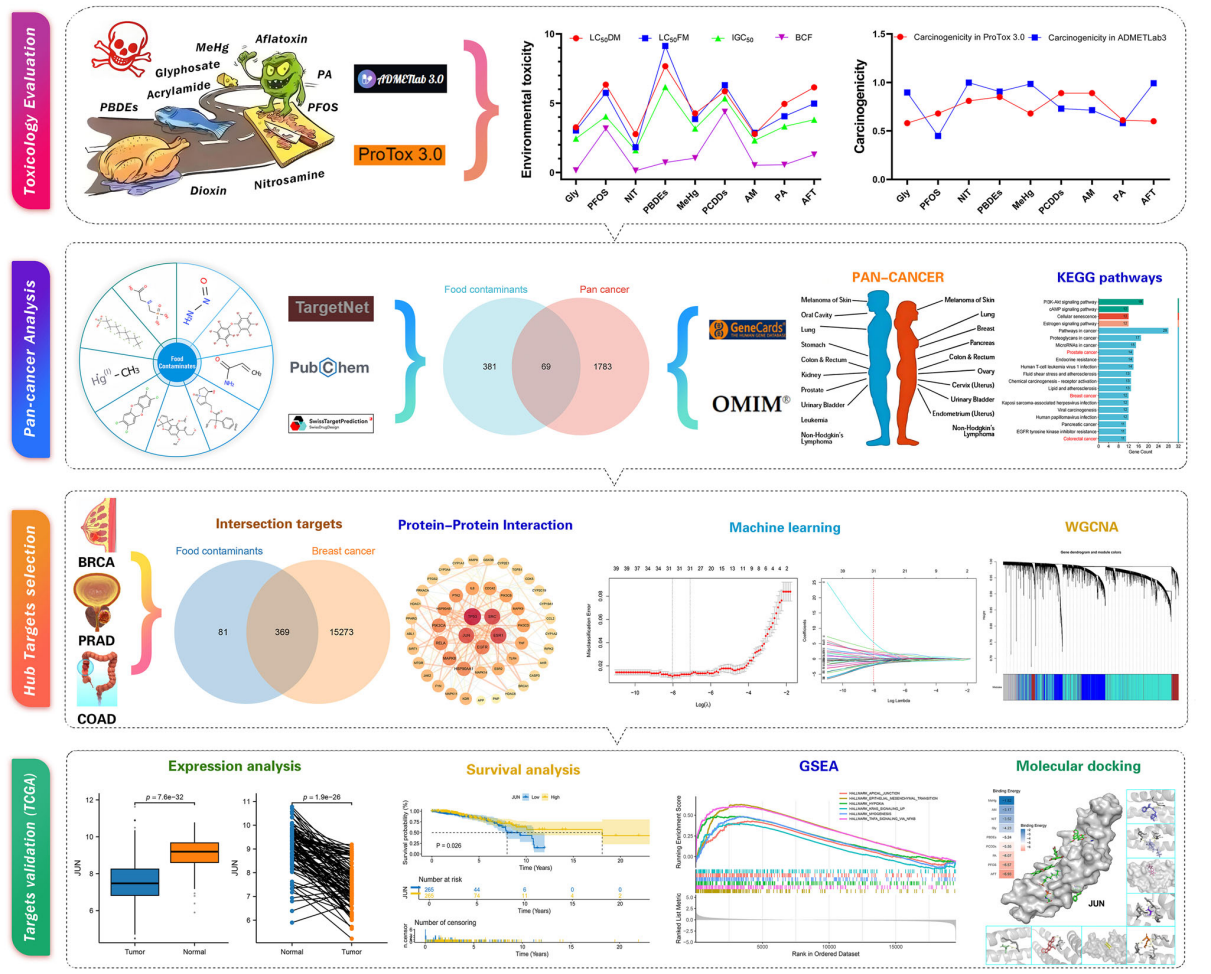
Workflow for this research.

This study presents an innovative multi‐modal approach integrating network toxicology, machine learning, WGCNA, and molecular docking to investigate foodborne carcinogenesis (Figure 1). Through systematic analysis of nine high‐risk contaminants, we identified pan‐cancer targets and key pathway modules, with subsequent validation of hub genes (JUN, CDC42, MAPK14) in breast, colon, and prostate cancers using TCGA datasets. Our approach not only delineates novel mechanisms but also provides actionable targets for therapeutic development. By linking molecular signatures to clinical outcomes, this work underscores the imperative of food safety reforms and precision public health strategies to mitigate the global cancer burden.

## 2 Methods

### 2.1 Identification of Physicochemical Properties and Toxicity of Food Contaminants

We obtained the chemical structures and molecular details of nine food contaminants from the PubChem database (Kim et al. 2022) (https://pubchem.ncbi.nlm.nih.gov). The carcinogenic properties of these pollutants were subsequently assessed using the ADMETLAB 3.0 platform (Fu et al. 2024) (https://admetlab3.scbdd.com, updated on January 31 2024) and the ProTox3 database (Banerjee, Kemmler, Dunkel, and Preissner 2024) (https://tox.charite.de/protox3, updated on May 2024), with the results summarized in Table 1.

**TABLE 1:** The physicochemical properties and toxicity assessment of compounds.

CAS	Compound term	Molecular formula	Molecular weight(g/mol)	LogP	LC_50_DM	LC_50_FM	IGC_50_	BCF	Carcinogenicity In ProTox 3.0	Carcinogenicity In ADMETLab3
1071‐83‐6	Glyphosate	C_3_H_8_NO_5_P	169.070	−3.419	3.259	3.032	2.439	0.150	0.58	0.896
1763‐23‐1	Perfluorooctane sulfonate	C_8_HF_17_O_3_S	500.130	1.817	6.341	5.744	4.039	3.178	0.68	0.448
35576‐91‐1	Nitrosamines	H_2_N_2_O	46.029	−0.617	2.769	1.826	1.603	0.130	0.81	0.998
1163‐19‐5	Pentabromodiphenyl ethers	C_12_Br_10_O	959.200	6.977	7.666	9.130	6.161	0.719	0.85	0.905
16056‐34‐1	Methyl mercury	CH_3_Hg	215.630	1.166	4.269	3.858	3.169	1.036	0.68	0.984
1746‐01‐6	Dioxins	C_12_H_4_Cl_4_O_2_	322.000	6.673	5.861	6.305	5.341	4.383	0.89	0.730
79‐06‐1	Acrylamide	C_3_H_5_NO	71.080	−0.513	2.779	2.861	2.307	0.522	0.89	0.714
81340‐07‐0	Pyrrolizidine alkaloid	C_18_H_23_NO_5_	333.400	0.630	4.953	4.054	3.318	0.560	0.61	0.580
1165‐39‐5	Aflatoxin	C_17_H_12_O_7_	328.270	1.654	6.146	4.973	3.809	1.296	0.60	0.992

“CAS”: Chemical Abstracts Service; “LogP”: The logarithm of the n‐octanol/water distribution coefficients at pH = 7.4; “BCF”: Bioconcentration factors; “LC_50_DM”: 50% lethal concentration in the daphnia magna after 48 h; “LC_50_FM”: 50% lethal concentration in the fathead minnow after 96 h; “IGC_50_”:  50% growth inhibition concentration in the tetrahymena pyriformis.

### 2.2 Collection of Food Contaminants Target Genes

The potential human target genes linked to the nine food contaminants were identified using data from four key databases: the STITCH database (Kuhn et al. 2008) (http://stitch.embl.de/cgi/), the Swiss Target Prediction database (Gfeller et al. 2014) (http://www.swisstargetprediction.ch/), the SEA database (Chen et al. 2020) (https://sea.bkslab.org/), and the TargetNet database (http://targetnet.scbdd.com). The gene data obtained from these four databases were consolidated, duplicates were eliminated, and a definitive set of target genes for the food contaminants was established.

### 2.3 Collection of Cancer‐Related Genes

The cancer‐related genes were obtained from two key databases, OMIM (Amberger, Bocchini, Schiettecatte, Scott, and Hamosh 2015) (https://omim.org/, accessed on March 25th, 2025) and GeneCards (Stelzer et al. 2016) (https://www.genecards.org/, accessed on March 25th, 2025), with selection criteria focusing on genes that surpassed a defined “score” threshold, specifically, the median score value from GeneCards. After merging the gene lists from both sources and eliminating duplicates, a final, non‐redundant collection of cancer‐associated genes was compiled.

### 2.4 The Intersection of Food Contaminants Targets and Cancer‐related Targets

The intersection analysis between contaminant targets and cancer‐related targets was conducted using the Venn diagram tool available on the web (http://www.bioinformatics.com.cn/). Both the contaminant target library and the cancer target library were uploaded to this platform, and the results were retrieved upon completion of the analysis.

### 2.5 Construction of Protein—Protein Interaction (PPI) Networks

We constructed the PPI network using the STRING database (Szklarczyk et al. 2021) (https://cn.string‐db.org/) by analyzing overlapping targets identified from the Venn diagram. The analysis was restricted to Homo sapiens, and we set a high‐confidence interaction threshold score of ≥0.9 to generate the initial PPI network. For further visualization and analysis, we imported the STRING data into Cytoscape 3.10.3 (Shannon et al. 2003), which allowed us to compute various topological properties and create a comprehensive network diagram. Core targets were selected based on nodes exceeding the median values for betweenness centrality, closeness centrality, and degree. The MCODE plugin was utilized to identify the most significant subclusters of interacting nodes, and the CytoHubba plugin was employed to predict the top 10 significant genes using the maximal clique centrality (MCC) algorithm. Hub genes were ascertained by intersecting the targets of the MCODE significant module with those predicted by CytoHubba.

### 2.6 GO and KEGG Pathway Analysis

To explore the pathways associated with cancer‐related interactome targets, we conducted Gene Ontology (GO) terms analysis, covering biological processes, molecular functions, and cellular components, as well as KEGG pathway analyses using the DAVID database (Sherman et al. 2022) (https://david.ncifcrf.gov/). This approach was designed to identify and highlight the key signaling pathways involved in these biological processes. For efficient interpretation and visualization of the GO and KEGG results, we utilized the platform available at https://www.bioinformatics.com.cn, which enabled clear and insightful representation of the findings.

### 2.7 Screening of Hub Targets Using Machine Learning Algorithms

We employed four machine learning algorithms: Lasso regression analysis, SVM‐RFE (support vector machine‐recursive feature elimination), random forest (Boruta feature selection), and Random Forest to screen potential targets within the key subnetwork. Using R Studio (version 4.4.2) with a fixed random seed of “123,” we analyzed the genes and identified overlapping candidates from these methods as potential toxicity‐related targets in cancers. To assess their clinical relevance, we developed a nomogram using the RMS package, which illustrates the 5‐year survival probability of cancer patients, highlighting the practical value of these interactome targets in clinical setting.

### 2.8 Identification of Potential Target Genes Through Weighted Gene Co‐Expression Network Analysis (WGCNA)

For the WGCNA analysis (Langfelder and Horvath 2008), we sourced cancerous and normal tissues from the UCSC Xena database. We confirmed the soft threshold using the pick Soft Threshold function from the WGCNA package in R software. After establishing a scale‐free network based on this soft threshold, a topology matrix and hierarchical clustering were applied. Subsequently, eigengenes were identified for each module. Based on these eigengenes, hierarchical clustering was performed once the correlation between modules was established. The module eigengene (ME), considered representative of the gene expression profiles, was calculated to identify modules associated with clinical traits. To pinpoint the most tumor‐related modules, we conducted Module‐Trait Relationships calculations for each module. Eigengene Adjacency Heatmaps were generated to illustrate module relationships.

### 2.9 Characterization of Key Targets for Expression, Survival Probability, and Gene Set Enrichment Analysis (GSEA)

The expression profiles of the identified key targets were correlated and visualized using the corrplot package within R Studio (version 4.4.3). The expression levels of each critical gene were assessed through the Wilcoxon rank sum test. Subsequently, GSEA was conducted for each key gene to gain further insights into the functions of the pathways enriched.

### 2.10 Molecular Docking

We obtained crystal structures of key targets by integrating data from the Protein Data Bank (PDB) and UniProt databases (UniProt 2023), preprocessing them with AutoDock Vina version v1.2.7 (Bugnon et al. 2024). The study specifically used PDB files for JUN (5FV8), MAPK14 (6SFO), and CDC42 (2QRZ). These structures were selected based on criteria including Homo sapiens origin and a resolution of less than 3.0 Å. The protein structures were prepared using PyMOL v2.3.0, which involved removing water molecules, adding polar hydrogen atoms, and assigning Kollman charges. The 3D structures of the small molecule ligands (food contaminants) were downloaded in SDF format from the PubChem database. Their geometric conformations were first optimized and energy‐minimized using Chem3D v15.0 (with the MMFF94 force field) to ensure stable initial states before docking. The search space for docking was defined by a grid box centered on the known active site of each protein target. The specific grid parameters, including the center coordinates and dimensions, were meticulously defined for each target to comprehensively encompass the entire binding cavity and allow for full ligand flexibility:

$$\begin{array}{ccc} & & \mathrm{JUN}\;\left(5\mathrm{FV}8\right):\mathrm{Grid}\;\mathrm{center}\;\left(x=31.631,y=7.991,z=10.901\right);\\ & & \;\mathrm{Grid}\;\mathrm{size}\left(x=44.58,y=33.88,z=74.90\right)\overset{˚}{A}. \end{array}$$

$$\begin{array}{ccc} & & \mathrm{MAPK}14\left(6\mathrm{SFO}\right):\;\mathrm{Grid}\;\mathrm{center}\;\left(x=-6.840,y=5.826,\right.\\ & & \;\left.z=-16.079\right);\;\mathrm{Grid}\;\mathrm{size}\;\left(x=72.00,y=52.00,z=46.00\right)\overset{˚}{A}. \end{array}$$

$$\begin{array}{ccc} & & \mathrm{CDC}42\left(2\mathrm{QRZ}\right):\mathrm{Grid}\;\mathrm{center}\;\left(x=41.677,y=13.078,\right.\\ & & \;\left.z=15.595\right);\mathrm{Grid}\;\mathrm{size}\;\left(x=74.00,y=72.00,z=58.00\right)\overset{˚}{A}. \end{array}$$

To ensure the reliability and consistency of the results, each ligand‐target pair was docked nine times. The conformation with the most favorable (lowest) binding affinity (reported in kcal/mol) from the generated poses was selected for subsequent interaction analysis. The resulting docking models were visualized and analyzed using PyMOL to examine key molecular interactions (e.g., hydrogen bonds, hydrophobic contacts). The binding energies for all pairs were systematically compared and represented as a heatmap generated using the pheatmap package in R Studio (version 4.4.3).

## 3 Results

### 3.1 Physicochemical and Toxicological Assessment of Contaminants

**FIGURE 2:**
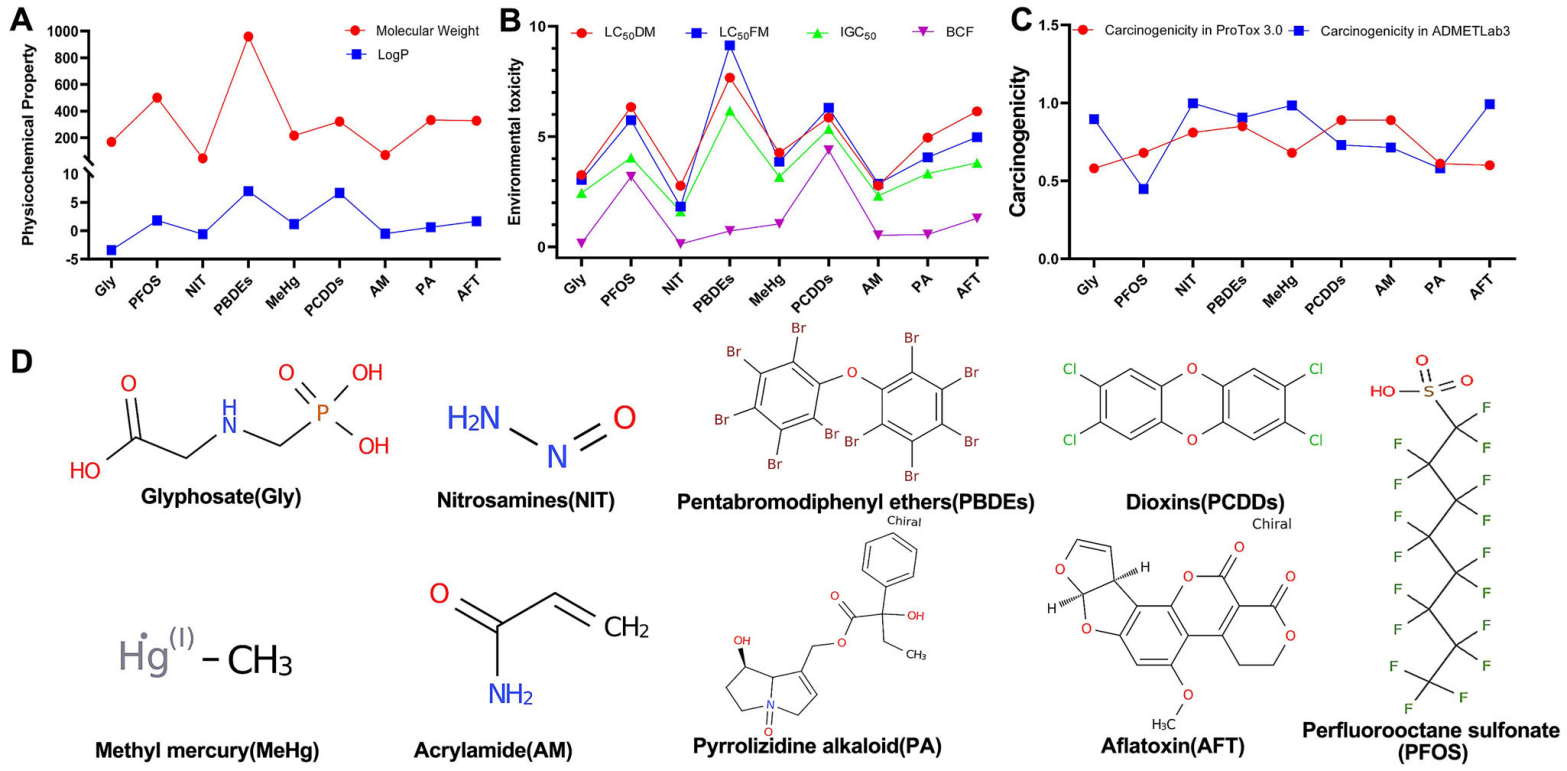
Comprehensive characterization and toxicological profiling of nine food contaminants. (A) Physicochemical properties of nine food contaminants, presenting calculated molecular weights (g/mol) and octanol‐water partition coefficients (Log P values). (B) Comparative ecotoxicological assessment including acute toxicity parameters (LC_50_DM: 50% lethal concentration in the daphnia magna after 48 h; LC_50_FM: 50% lethal concentration in the fathead minnow after 96 h; IGC_50_: 50% growth inhibition concentration in the tetrahymena pyriformis; BCF: bioconcentration factors are used for considering secondary poisoning potential and assessing risks to human health via the food chain). (C) The carcinogenic potential of these contaminants was assessed in both ProTox 3.0 and ADMETlab 3 platforms. (D) Chemical structure formulas of the nine amanitins.

We conducted a comprehensive assessment of nine representative contaminants by systematically analyzing their physicochemical properties and toxicological profiles using ADMET and ProTox approaches (Figure 2). Our analysis revealed a positive correlation trend between molecular weight and lipophilicity among these contaminants (Figure 2A). To evaluate environmental toxicity, we measured lethal concentrations (LC_50_) against three model organisms: Tetrahymena thermophila, Pimephales promelas, and Daphnia magna, which showed varying bioconcentration factors across the contaminants (Figure 2B). Importantly, both ProTox 3.0 and ADMETlab 3 platforms indicated significant carcinogenic potential for these substances, with values generally exceeding 0.5 (except for PFOS, which has a value of 0.448 in ADMETLab3 and 0.68 in ProTox 3.0) (Figure 2C). These results align with established clinical data on the toxicity of these contaminants (Hardell et al. 2023; Rhee et al. 2023; Yu et al. 2023), further validating their particularly strong carcinogenic effects.

### 3.2 Identification of Food Contaminant‐Pan‐Cancer Core Targets and PPI Network Construction

**FIGURE 3:**
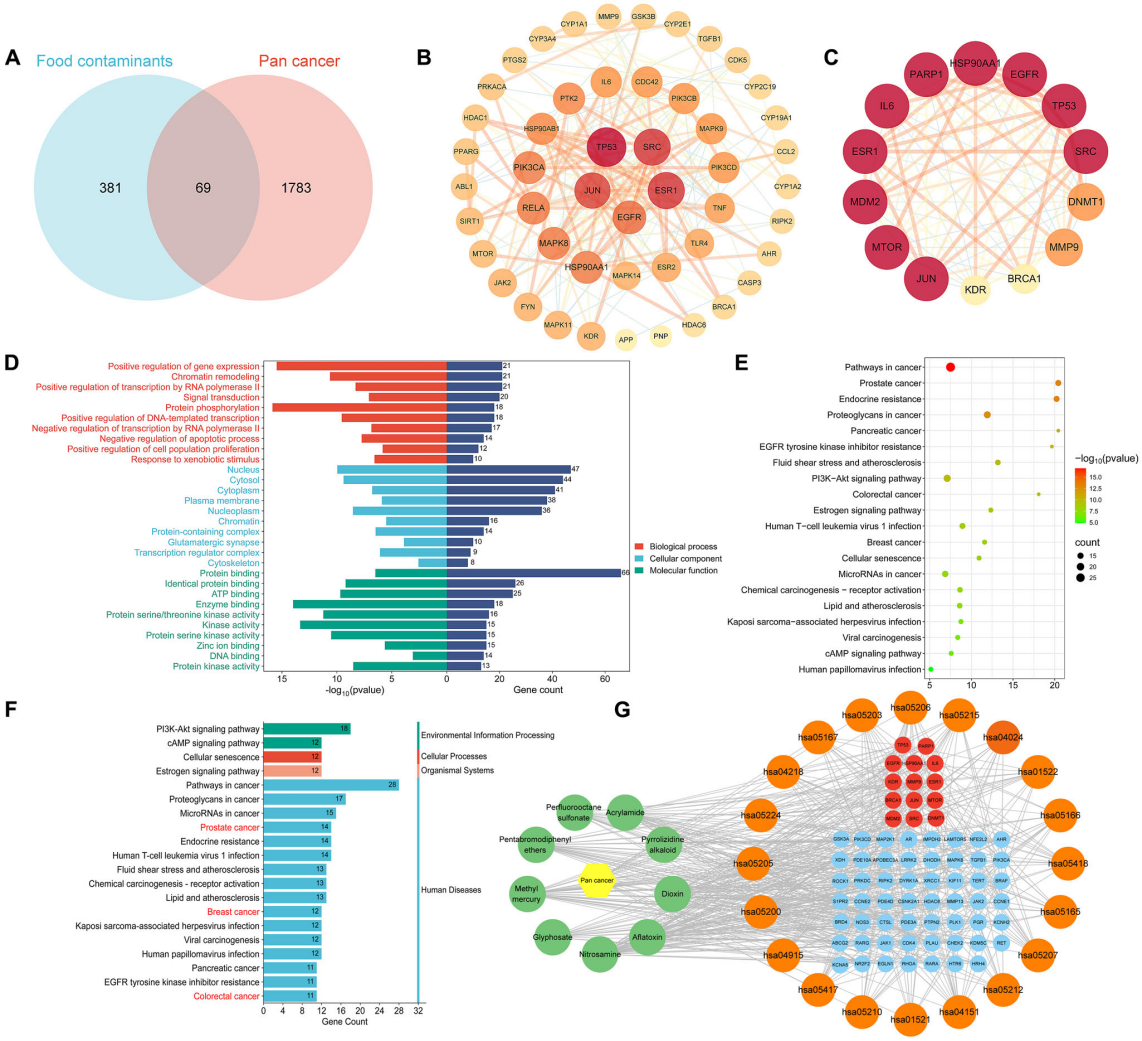
The associations between food contaminants and pan‐cancer. (A) Venn diagram depicting the overlap of targets associated with food contaminants and pan cancer. (B) Visualization of interacting targets using Cytoscape 3.10.3, illustrating the network of target interactions. (C) Potential hub genes are arranged in descending order of degree value starting from the highest degree value. The red color intensity of each node represents its degree, with a deeper red indicating a higher value. (D) GO enrichment analysis highlighting significant biological processes, cellular components, and molecular functions related to the interact targets. (E, F) KEGG enrichment analysis of the interact targets, identifying enriched pathways. (G) Compound‐target‐pathway network diagram of food contaminants induced pan cancer.

We screened 450 food contaminant targets from multiple databases (STITCH, Swiss Target Prediction, SEA, and TargetNet), cross‐referenced with 1,852 pan‐cancer targets from GeneCards and OMIM, and identified 69 potential targets linked to contaminant‐induced pan‐cancer (Figure 3A). Using STRING, we constructed PPI networks and refined them in Cytoscape using centrality criteria: Closeness unDir > 0.007993356128408048, Betweenness unDir > 63.617647058823486, and Degree unDir > 17.676470588235293, identifying 14 core targets (Figure 3B,C).

GO and KEGG enrichment analyses identified 130 KEGG pathways and 276 GO terms, including 167 biological processes (BP), 22 cellular components (CC), and 87 molecular functions (MF). The top 10 GO entries with the highest gene ratios were highlighted in a bar chart (Figure 3D), showing key processes like positive regulation of gene expression, chromatin remodeling, and positive regulation of transcription by RNA polymerase II. The top 20 KEGG entries were plotted (Figure 3E,F), highlighting cancer‐related pathways like pathways in cancer, PI3K‐Akt signaling pathway, Proteoglycans in cancer. We selected three cancers for further analysis. A contaminant‐target‐pathway network constructed in Cytoscape illustrated interactions among 9 environmental contaminants (green nodes), 20 biological pathways (orange nodes), associated target genes (blue nodes), and 14 potential hub genes (red nodes) (Figure 3G).

### 3.3 The Effect of Food Contaminants on BRCA

**FIGURE 4:**
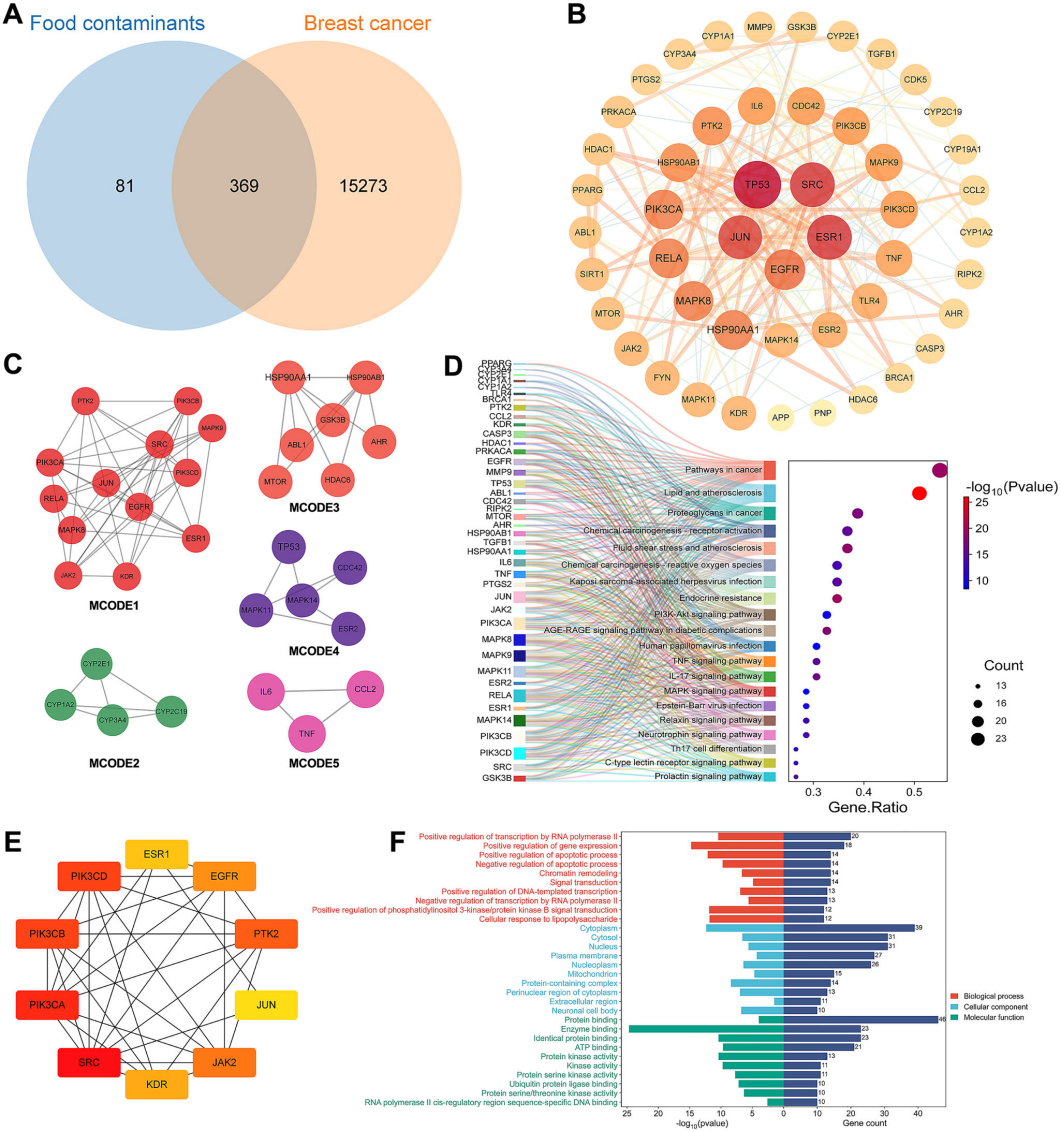
Analysis of associations between food contaminants and breast cancer. (A) The Venn diagram of related targets between food contaminants and breast cancer. (B) PPI network diagram of core targets. (C) MCODE classification of core targets. (D) The top 20 KEGG enrichment results displayed in gene ratio order and the relationship between the select core genes and pathways. (E) Top 10 Hubba gene of core targets. (F) GO enrichment results.

We integrated 15,632 breast cancer‐related targets with 450 food contaminant targets, identifying 369 potential targets associated with contaminant‐induced breast cancer (Figure 4A). A high‐confidence (score ≥0.9) PPI network constructed via STRING and analyzed in Cytoscape identified 49 core targets using centrality criteria: Closeness >0.0010247338113175926, Betweenness >758.5291828793742, and Degree >6.64591439688716 (Figure 4B). Subsequent MCODE analysis clustered these targets into five modules (Figure 4C), while the CytoHubba revealed the top 10 highest‐degree targets (Figure 4E).

GO and KEGG enrichment analyses identified 161 KEGG pathways and 634 GO terms, which included 441 BP, 51 CC, and 142 MF. The BP category included transcription regulation by RNA polymerase II, positive gene expression regulation, apoptosis regulation, and chromatin remodeling. The CC category related to cytoplasm, cytosol, and nucleus, while MF concerned protein binding, enzyme binding, and identical protein binding (Figure 4F). Additionally, KEGG pathway analysis demonstrated significant enrichment in cancer‐related pathways, including pathways in cancer, proteoglycans in cancer, and chemical carcinogenesis‐receptor activation (Figure 4D).

**FIGURE 5:**
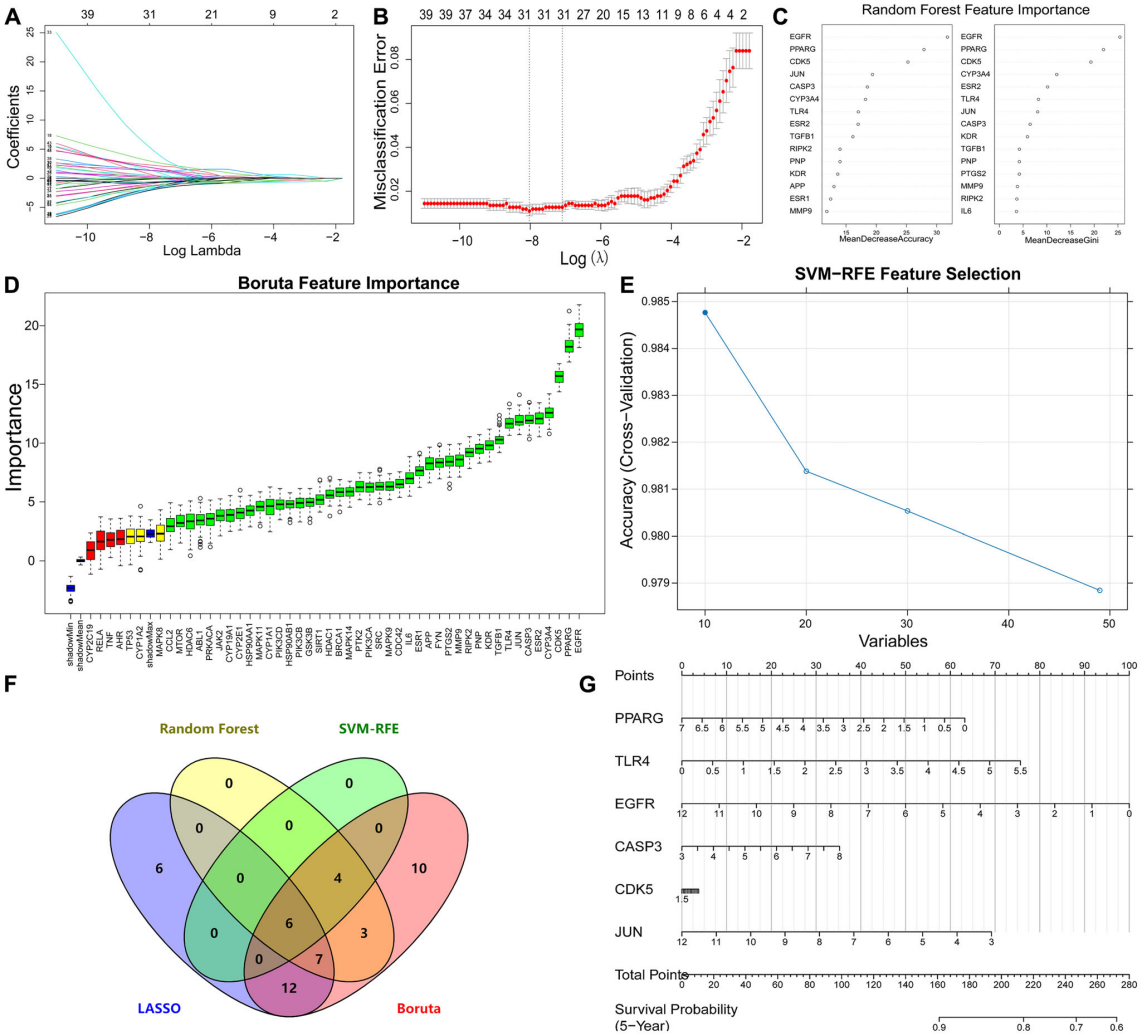
Machine learning‐based screening of key targets. (A) LASSO coefficient profiles. The LASSO model was applied for regression of high‐dimensional predictors. The method uses an L1 penalty to shrink some regression coefficients to exactly zero, thereby selecting the most significant predictors. Each curve represents the trajectory of a predictor's coefficient as λ varies. (B) Lasso coefficient distribution misclassification error. (C) Feature importance of the random forest model. (D) Boruta‐based feature selection was used to sort the importance of the features. (E) SVM‐RFE model. (F) Venn diagram of four algorithms for screening key targets. (G) The nomogram is based on the six‐gene signature.

Machine learning feature screening was executed by constructing LASSO regression with an optimal λ value of 0.0003276885, identifying 31 genes (Figure 5A,B). The Random forest, Boruta, and SVM‐RFE algorithms were applied further refining signature genes (Figure 5C–E). By intersecting the signature genes obtained from these four models, six genes (PPARG, TLR4, EGFR, CASP3, CDK5, JUN) were pinpointed as potential key targets for subsequent analysis (Figure 5F). A nomogram developed using the Rms package was used to estimate 5‐year survival probabilities in breast cancer patients, demonstrating their clinical relevance (Figure 5G).

We applied WGCNA to the TCGA‐BRCA dataset, using a soft threshold power of 10 to define co‐expression modules, identifying 17 distinct gene clusters (Figure S1A–C). Module‐trait correlation analysis showed the green‐yellow (coefficient 0.46) and grey modules (coefficient 0.5) as positively associated with tumor development, while the brown module correlated with normal tissue (coefficient 0.66) (Figure S1D). Eigengene adjacency heatmaps depicting these relationships are illustrated in Figure S2E. By interacting with the hub genes that were identified through machine learning, two targets, JUN and EGFR, were identified.

**FIGURE 6:**
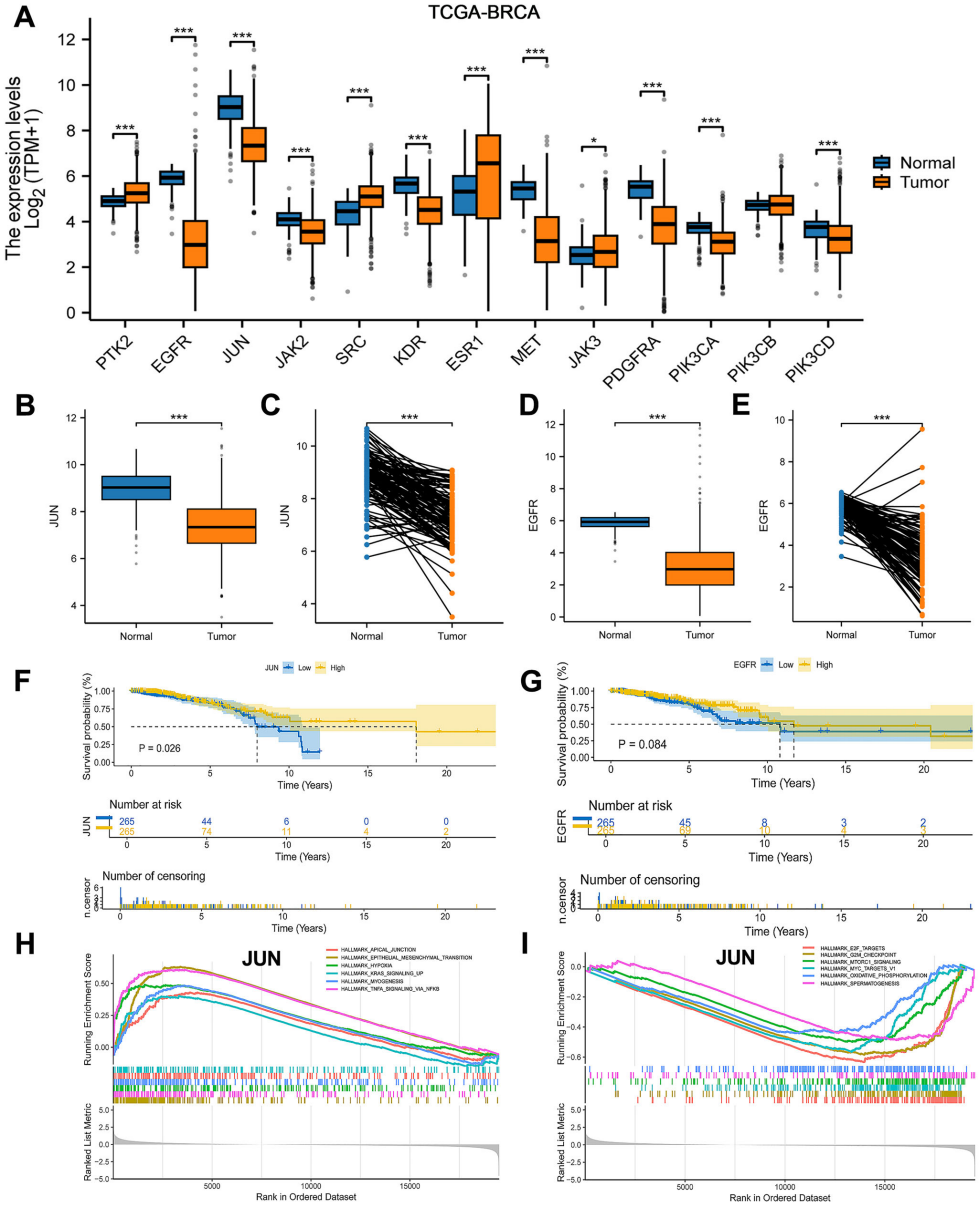
Characterization of hub targets for expression, survival probability, and gene set enrichment analysis. (A) Expression of hub targets in tumors and normal tissues in the TCGA‐BRCA dataset, and Wilcoxon rank sum was used for the significance test. (B, D) Differentiated expression of JUN and EGFR in the tumor and normal sample. Analyses were performed across all normal and tumor samples with p value closing to zero by Wilcoxon rank sum test. (C, E) Paired differentiation analysis for expression of JUN and EGFR in the normal and tumor sample deriving from the same one patient. (F, G) Survival analysis for BRCA patients with different targets expression. Patients were labeled with higher quartile versus lower quartile groups defined by expression of each gene indicated. *P*‐value was examined by log‐rank test. (H, I) GSEA enrichment results for JUN.

RNA sequencing (RNA‐seq) data from BRCA and GTEX cohorts within TCGA (comprising 179 normal and 1,099 tumor samples), were log2‐transformed for expression analysis (Figure 6A). The Wilcoxon rank sum test demonstrated significantly lower expression levels of JUN and EGFR in tumor samples compared to normal tissues, a finding further validated by paired sample comparisons (Figure 6B–E). Survival analysis revealed that BRCA patients with JUN expression in the higher quartile exhibited longer survival times than those with lower quartile expression (Figure 6F,G). GSEA of JUN identified several functionally relevant pathways, such as apical junction formation, EMT(epithelialmesenchymal transition), hypoxia response, E2F targets, and G2M checkpoint regulation (Figure 6H,I).

**FIGURE 7:**
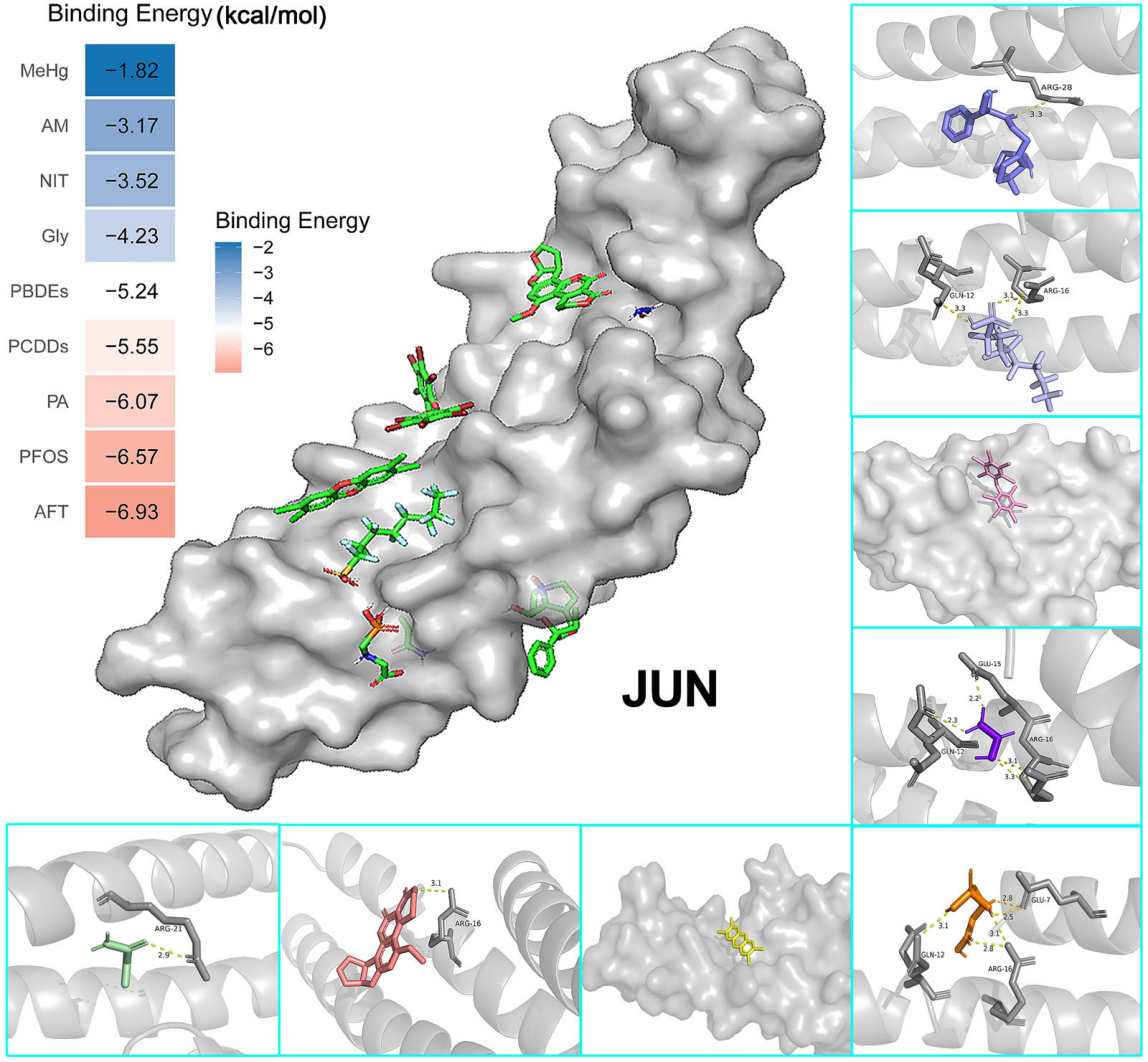
Molecular docking results of the lowest binding energy in nine food contaminants with the JUN.

Molecular docking of JUN with nine food contaminants demonstrated strong binding affinities, with the lowest binding energy reaching −6.93 kcal/mol (Figure 7). Key amino acid residues in JUN, such as ARG‐21, ARG‐16, GLN‐12, ARG‐28, GLU‐7, and GLU‐15, were found to participate in these binding events, suggesting potential functional implications of the contaminants on JUN protein activity.

### 3.4 The Effect of Food Contaminants on PRAD

**FIGURE 8:**
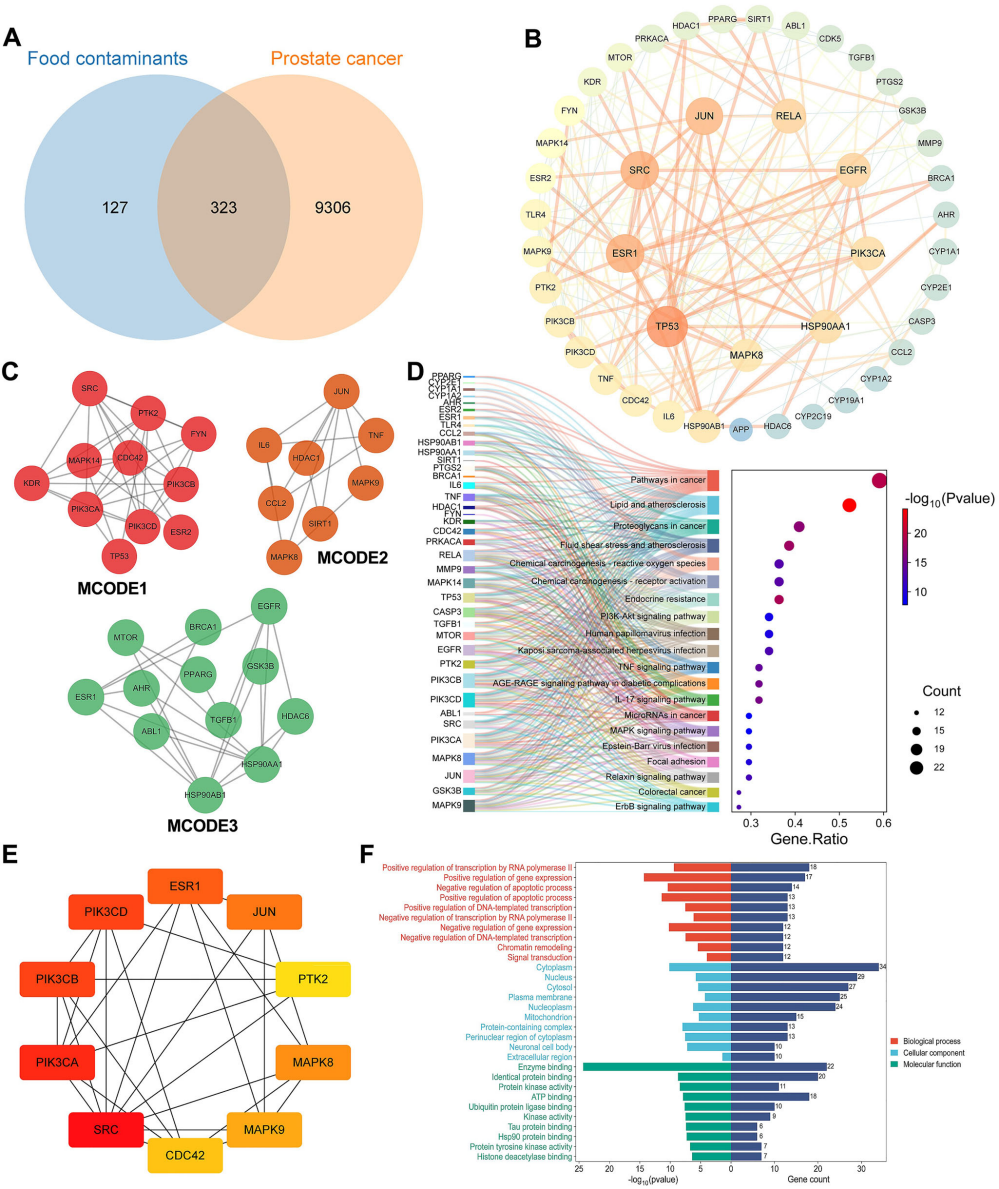
Analysis of associations between food contaminants and prostate cancer. (A) The Venn diagram of related targets between food contaminants and prostate cancer. (B) PPI network diagram of core targets. (C) MCODE classification of core targets. (D) The top 20 KEGG enrichment results displayed in gene ratio order and the relationship between the select core genes and pathways. (E) Top 10 Hubba gene of core targets. (F) GO enrichment results.

We integrated 9,629 prostate cancer‐related targets with 450 food contaminant targets, identifying 323 overlapping candidates (Figure 8A). Protein interaction networks were constructed using the STRING database with a high‐confidence interaction score cutoff of 0.9 and analyzed in Cytoscape pinpointed 44 key targets using centrality criteria: Closeness >0.0011772220504692277, Betweenness >650.0086206896538, and Degree >6.793103448275862 (Figure 8B). MCODE clustering grouped targets into three prostate cancer‐related modules (Figure 8C), while CytoHubba ranked the top 10 most interconnected targets (Figure 8E).

DAVID analysis identified 163 KEGG pathways and 606 GO terms, comprising 415 BP, 53 CC, and 138 MF. Top enriched terms included regulation of transcription by RNA polymerase II, regulation of gene expression, regulation of apoptotic process, and regulation of DNA‐templated transcription (BP); cytoplasm, nucleus, and cytosol (CC), while MF concerned enzyme binding, identical protein binding, and protein kinase activity (Figure 8F). KEGG pathway analysis indicated significant involvement in cancer pathways, including pathways in cancer, proteoglycans in cancer, and chemical carcinogenesis‐receptor activation (Figure 8D).

**FIGURE 9:**
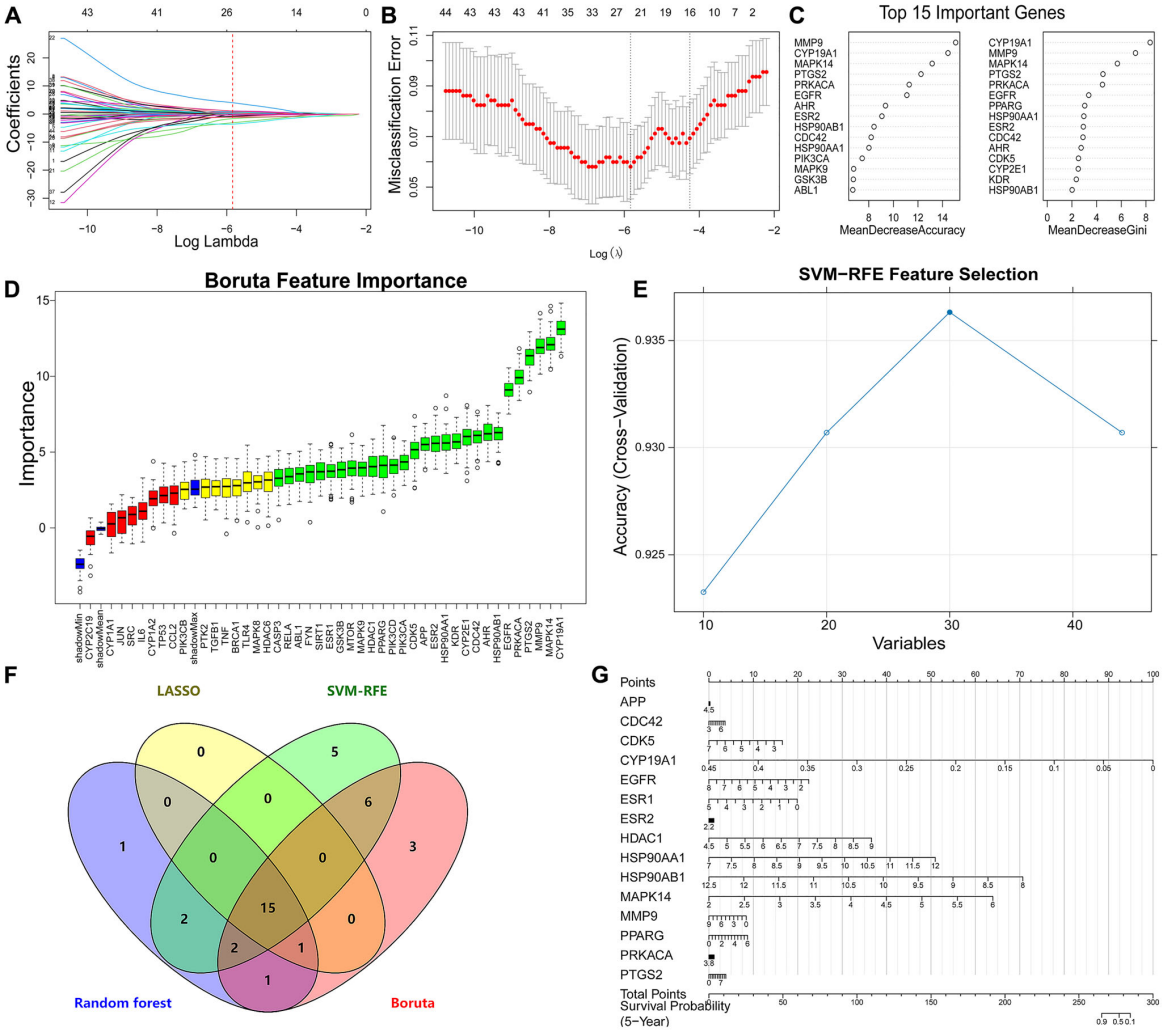
Machine learning‐based screening of key targets. (A) LASSO coefficient profiles. The LASSO model was applied for regression of high‐dimensional predictors. (B) Lasso coefficient distribution misclassification error. (C) Feature importance of the random forest model. (D) Boruta‐based feature selection was used to sort the importance of the features. (E) SVM‐RFE model. (F) Venn diagram of four algorithms for screening key targets. (G) The nomogram is based on the 15‐gene signature.

Machine learning identified 22 significant genes through LASSO regression with an optimal λ value of 0.00291347 (Figure 9A,B). Additionally, the SVM‐RFE algorithm selected 30 optimal candidates (Figure 9E). Further refinement was achieved through random forest and Boruta algorithms, which helped narrow down potential signature genes (Figure 9C,D); Integration of these four methods yielded 15 consensus genes: APP, CDC42, CDK5, CYP19A1, EGFR, ESR1, ESR2, HDAC1, HSP90AA1, HSP90AB1, MAPK14, MMP9, PPARG, PRKACA, and PTGS2 (Figure 9F). To highlight their clinical relevance, we developed a nomogram using the Rms package to predict the 5‐year survival probability for prostate cancer patients (Figure 9G).

WGCNA analysis of TCGA‐PRAD data utilized a soft threshold power of 10, generating 14 gene modules (Figure S2A–C). By examining module‐trait relationships, we found that the black and turquoise modules showed significant positive correlations with tumor development (both with coefficients of 0.32), while the pink (coefficient 0.41) and magenta (coefficient 0.45) modules were strongly associated with normal tissue conditions (Figure S2D). These relationships were further visualized through eigengene adjacency heatmaps (Figure S2E). Integration of these results with the machine learning approaches identified MAPK14 and CDC42 as potential key regulators in prostate cancer progression.

**FIGURE 10:**
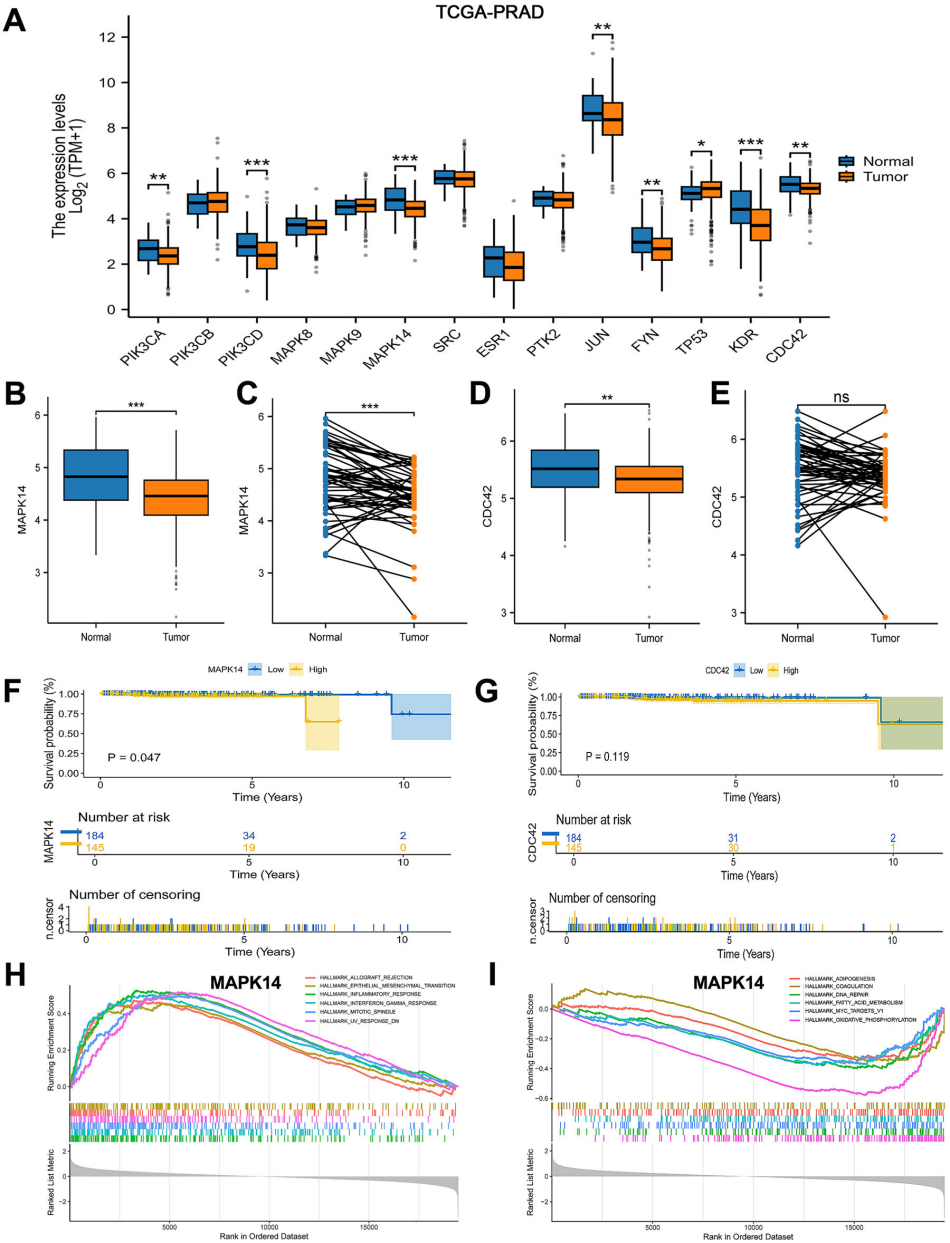
Characterization of hub targets for expression, survival probability, and gene set enrichment analysis. (A) Expression of hub targets in tumors and normal tissues in the TCGA‐PRAD dataset, and Wilcoxon rank sum was used for the significance test. (B, D) Differentiated expression of MAPK14and CDC42 in the tumor and normal sample. Analyses were performed across all normal and tumor samples with p value closing to zero by Wilcoxon rank sum test. (C, E) Paired differentiation analysis for expression of MAPK14and CDC42 in the normal and tumor sample deriving from the same one patient. (F, G) Survival analysis for PRAD patients with different targets expression. Patients were labeled with higher quartile versus lower quartile groups defined by expression of each gene indicated. P‐value was examined by log‐rank test. (H, I) GSEA enrichment results for MAPK14.

RNA‐seq data in TPM format from PRAD and GTEX cohorts within TCGA (comprising 100 normal and 496 tumor samples), were log2‐transformed for expression analysis (Figure 10A). The Wilcoxon rank sum test showed significantly lower MAPK14 and CDC42 expression in tumors versus normal tissues (Figure 10B,D). Paired analysis of matched normal‐tumor samples confirmed this pattern for MAPK14 but not CDC42 (Figure 10C,E). Survival analysis demonstrated that PRAD patients with MAPK14 expression in the lowest quartile had better survival outcomes than those with higher expression (Figure 10F,G). GSEA revealed MAPK14's involvement in key pathways including allograft rejection, EMT, inflammation, adipogenesis, DNA repair, and coagulation (Figure 10H,I).

**FIGURE 11:**
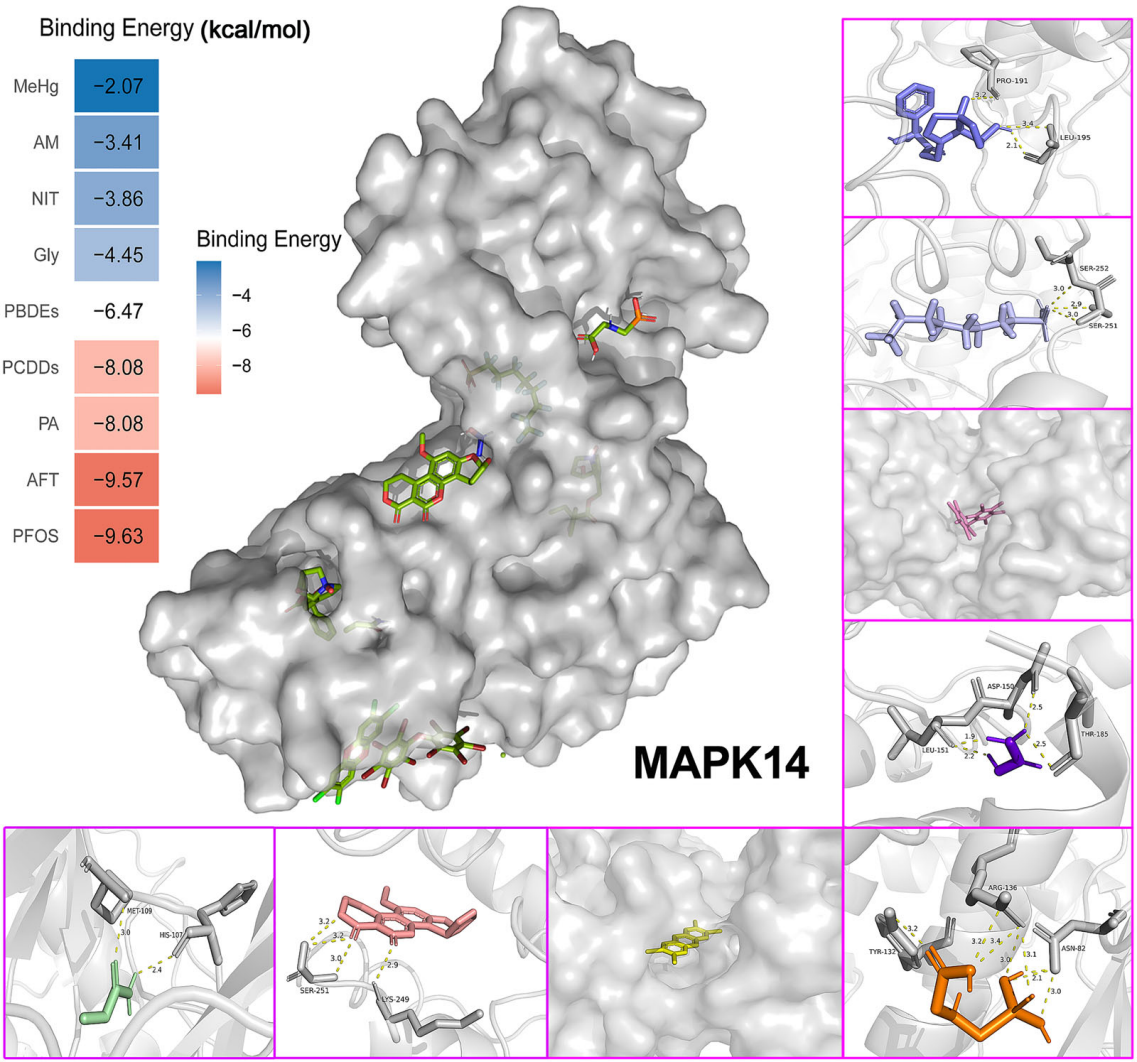
Molecular docking results of the lowest binding energy in nine food contaminants with the MAPK14.

Molecular docking of MAPK14 with nine food contaminants demonstrated strong binding affinities, with the lowest binding energy reaching −9.63 kcal/mol (Figure 11). Key amino acid residues in MAPK14 included MET‐109, HIS‐107, SER‐251, SER‐252, LYS‐249, TYR‐132, ARG‐136, ASN‐82, LEU‐151, ASP‐150, THR‐185, PRO‐191, and LEU‐195, suggesting potential binding sites.

### 3.5 The Effect of Food Contaminants on COAD

**FIGURE 12:**
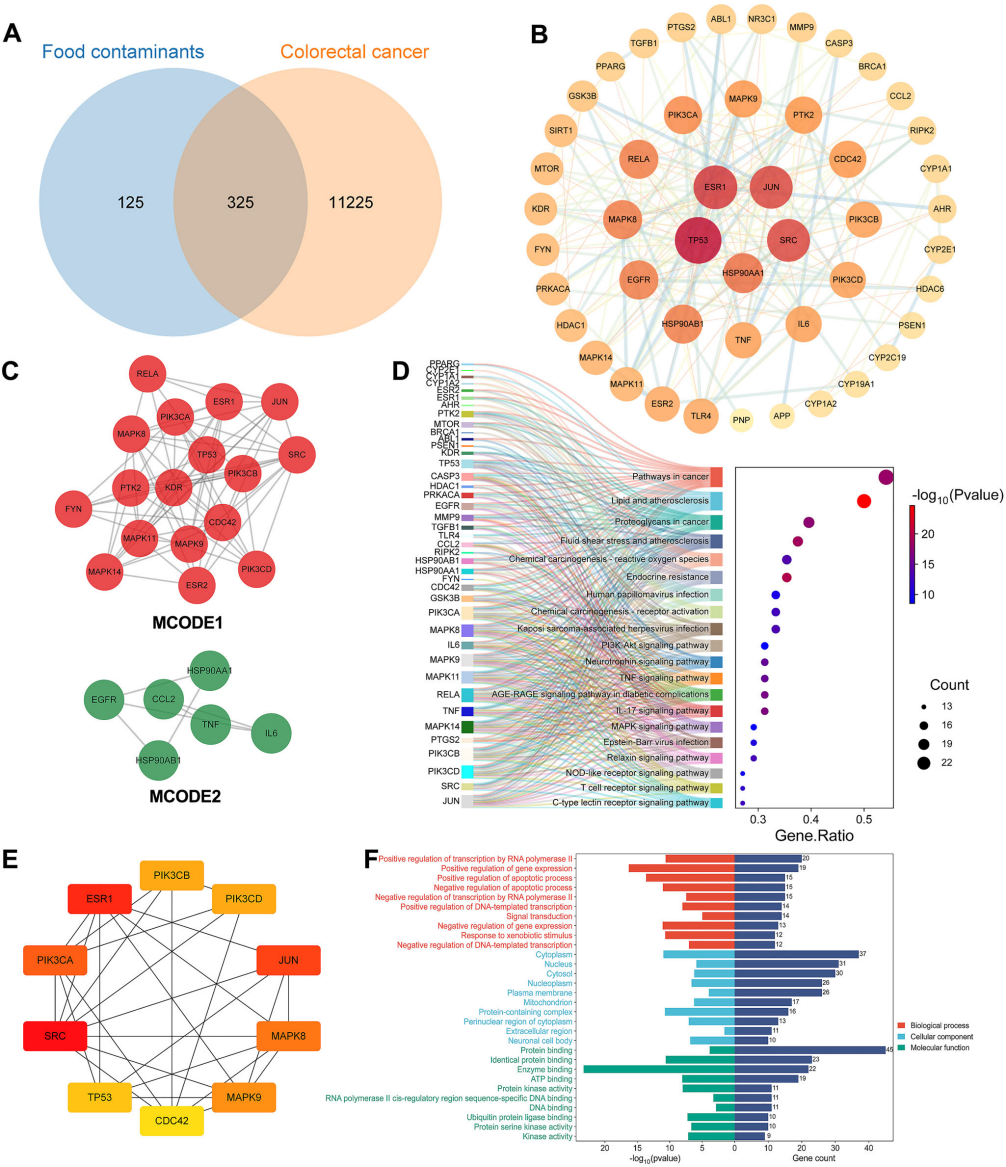
Analysis of associations between food contaminants and colorectal cancer. (A) The Venn diagram of related targets between food contaminants and colorectal cancer. (B) PPI network diagram of core targets. (C) MCODE classification of core targets. (D) The top 20 KEGG enrichment results displayed in gene ratio order and the relationship between the select core genes and pathways. (E) Top 10 Hubba gene of core targets. (F) GO enrichment results.

Analysis of 11,550 potential colorectal cancer targets from GeneCards and OMIM databases and 450 food contaminant targets identified 325 overlapping targets that potentially link food contaminants to colorectal cancer (Figure 12A). A PPI network was constructed using the STRING database with a high‐confidence interaction score cutoff of 0.9, and analyzed in Cytoscape. We identified 48 key targets by applying specific network metrics: closeness centrality >0.0012390264023192084, betweenness centrality >611.297777777777, and degree centrality >6.844444444444444 (Figure 12B). The MCODE plugin clustered these targets into two colorectal cancer‐related modules (Figure 12C), while CytoHubba analysis revealed the top 10 highest‐degree targets (Figure 12E).

GO and KEGG enrichment analysis using the DAVID database yielded 162 KEGG entries and 630 GO entries, including 434 BP, 55 CC, and 141 MF. The BP category was associated with regulation of transcription by RNA polymerase II, regulation of gene expression, regulation of apoptotic process, and regulation of DNA‐templated transcription. The CC category related to cytoplasm, nucleus, and cytosol, while MF concerned protein binding, identical protein binding, enzyme binding, and ATP binding (Figure 12F). KEGG pathway analysis indicated significant involvement in cancer pathways, including pathways in cancer, proteoglycans in cancer, and chemical carcinogenesis—reactive oxygen species (Figure 12D).

**FIGURE 13:**
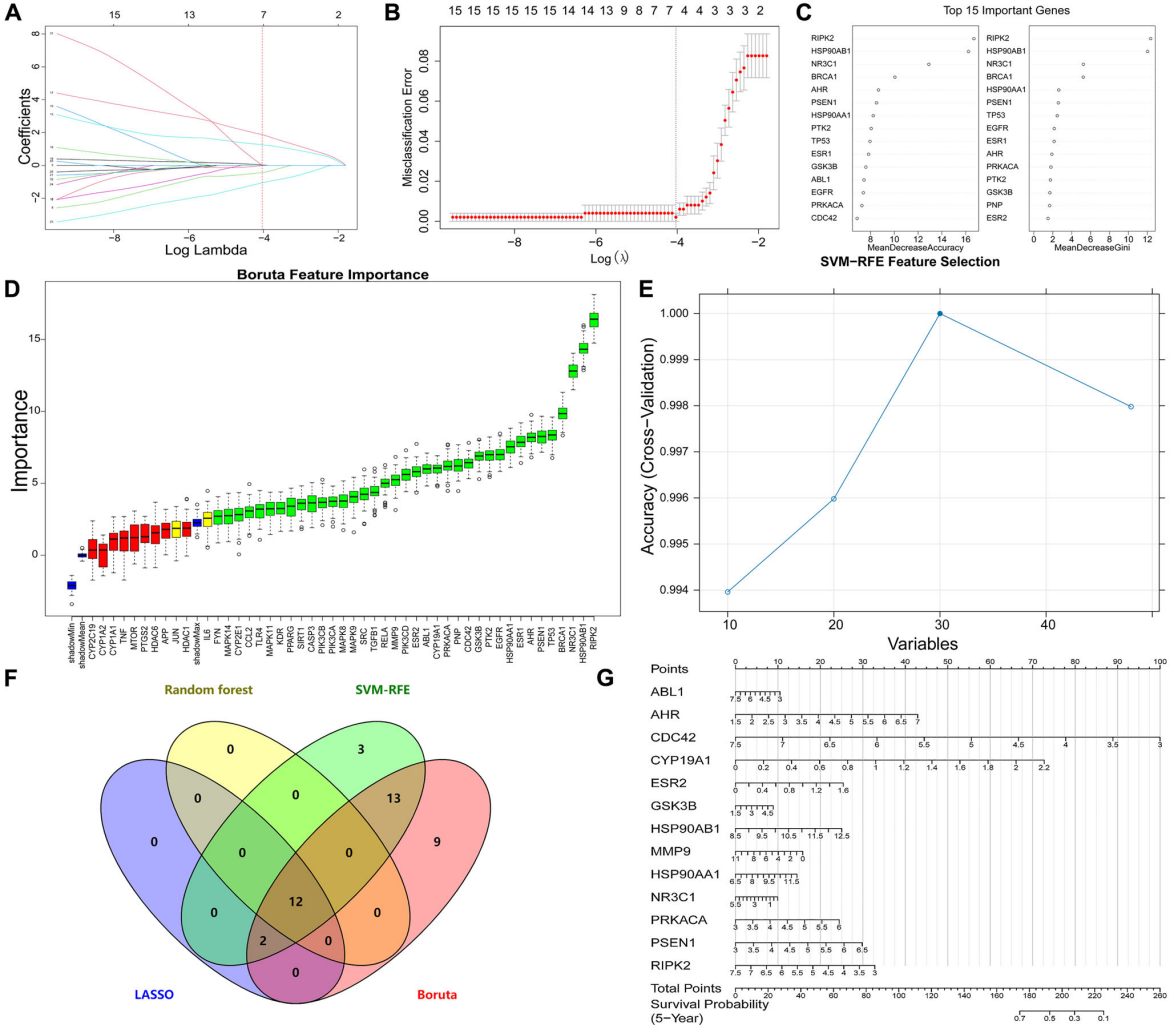
Machine learning‐based screening of key targets. (A) LASSO coefficient profiles. The LASSO model was applied for regression of high‐dimensional predictors. (B) Lasso coefficient distribution misclassification error. (C) Feature importance of the random forest model. (D) Boruta‐based feature selection was used to sort the importance of the features. (E) SVM‐RFE model. (F) Venn diagram of four algorithms for screening key targets. (G) The nomogram is based on the 15‐gene signature.

A comprehensive machine learning approach identified 14 candidate genes through LASSO regression with an optimal λ value of 0.01774578 (Figure 13A,B). The SVM‐RFE algorithm further refined the selection to 30 optimal candidates (Figure 13E). We then employed random forest classification alongside Boruta feature importance algorithms to validate potential signature genes (Figure 13C,D). Through integration of results from all four methods, we consistently identified twelve critical genes: ABL1, AHR, CDC42, CYP19A1, ESR2, GSK3B, HSP90AB1, MMP9, NR3C1, PRKACA, PSEN1, and RIPK2 (Figure 13F). A predictive nomogram using the Rms package estimated 5‐year survival probabilities for colorectal cancer patients, providing a practical tool for clinical assessment (Figure 13G).

WGCNA analysis of TCGA‐COAD dataset identified key gene modules using an optimal soft threshold power of 10 for network construction, as shown in supplementary Figures S3A and S3B. The analysis revealed 21 distinct gene modules through dynamic tree cutting (Figure S3C). When examining module‐trait relationships, we found that the lightcyan (coefficient 0.57) and magenta (coefficient 0.38) modules showed significant positive associations with tumor development, while the yellow (coefficient 0.78) and turquoise (coefficient 0.72) modules were strongly correlated with normal tissue conditions (Figure S3D). These relationships were further visualized through eigengene adjacency heatmaps (Figure S3E). Integration with machine learning‐selected hub genes identified ESR2 and CDC42 as promising molecular targets potentially underlying tumor and normal tissues differences in colorectal adenocarcinoma.

**FIGURE 14:**
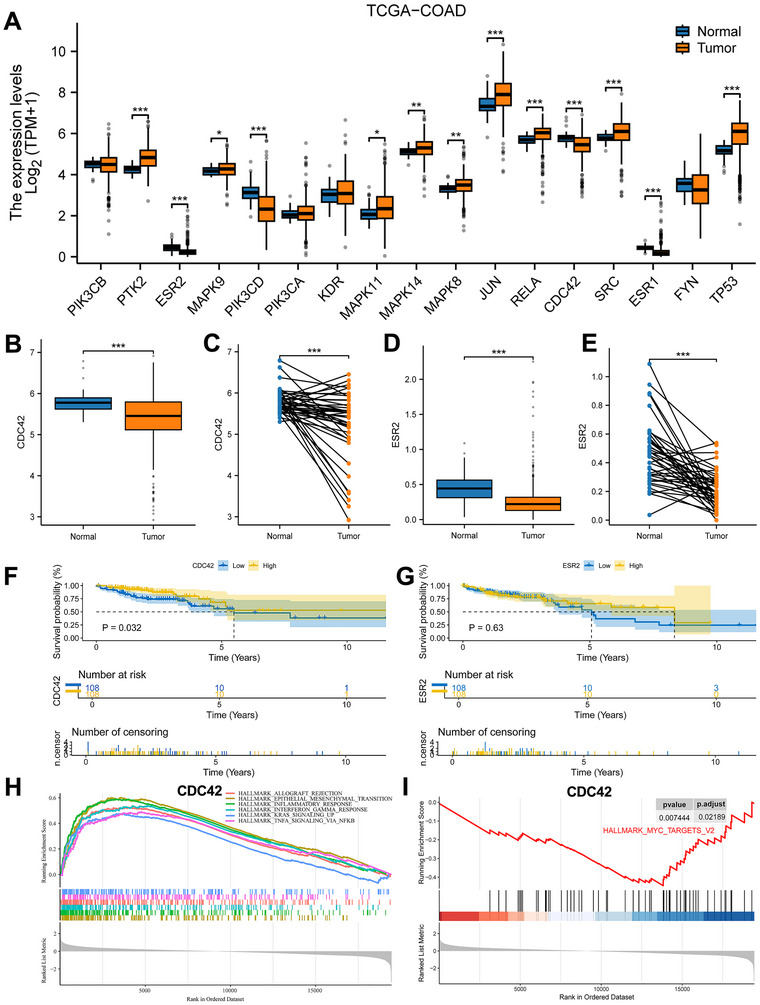
Characterization of hub targets for expression, survival probability, and gene set enrichment analysis. (A) Expression of hub targets in tumors and normal tissues in the TCGA‐PRAD dataset, and Wilcoxon rank sum was used for the significance test. (B, D) Differentiated expression of MAPK14and CDC42 in the tumor and normal sample. Analyses were performed across all normal and tumor samples with *p* value closing to zero by Wilcoxon rank sum test. (C, E) Paired differentiation analysis for expression of MAPK14and CDC42 in the normal and tumor sample deriving from the same one patient. (F, G) Survival analysis for PRAD patients with different targets expression. Patients were labeled with higher quartile versus lower quartile groups defined by expression of each gene indicated. *P*‐value was examined by log‐rank test. (H, I) GSEA enrichment results for CDC42.

RNA‐seq data in the TPM format from COAD and GTEX cohorts within TCGA (comprising 308 normal and 290 tumor samples), was log2‐transformed for expression analysis (Figure 14A). The Wilcoxon rank sum test revealed significantly lower expression levels of CDC42 and ESR2 in tumor samples compared to normal tissues. This trend was further confirmed in paired analyses of normal and tumor tissues from the same patients (Figure 14B–E). Survival analysis demonstrated that COAD patients with CDC42 expression in the lower quartile had reduced survival probability compared to those with higher CDC42 expression, while ESR2 expression did not show a significant survival difference (Figure 14F,G). GSEA of CDC42 highlighted several relevant pathways, such as allograft rejection, epithelial‐mesenchymal transition, inflammatory response, interferon gamma response, and MYC targets (Figure 14H,I).

**FIGURE 15:**
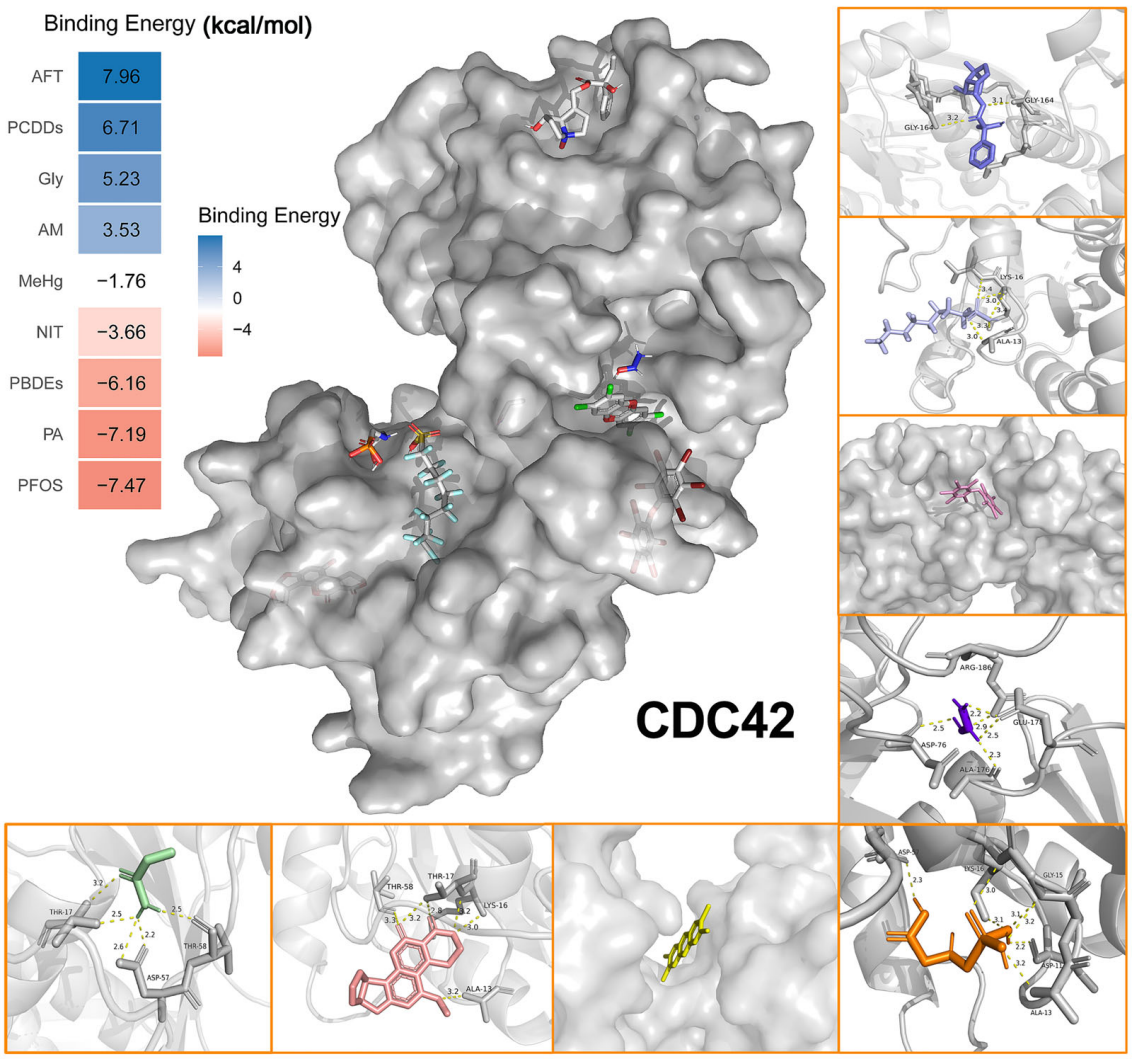
Molecular docking results of the lowest binding energy in 9 food contaminants with the CDC42.

Molecular docking analysis of CDC42 with food contaminants demonstrated strong binding affinities, with the lowest binding energy reaching −7.47 kcal/mol (Figure 15). These contaminants were found to engage with critical amino acid residues of CDC42, such as THR‐17, THR‐58, ASP‐57, ASP‐11, ASP‐176, LYS‐16, ALA‐13, ALA‐176, ARG‐186, GLY‐15, GLU‐178, and GLY‐164.

## 4 Discussion

The widespread presence of contaminants in our global food supply has raised serious concerns about their harmful effects on human health, especially their potential to cause cancer (Rushing and Selim 2019). Although extensive research has been conducted on the toxicological profiles of food contaminants like Glyphosate, PFOS, Nitrosamines, and others (Alikord et al. 2022; Soares et al. 2021; Zhang et al. 2024), the molecular pathways through which these agents promote the development of cancer remain inadequately elucidated. In this study, we employed a comprehensive array of advanced databases and bioinformatics network tools, including PubChem, ADEMTlab3.0, Protox3, GeneCards, OMIM, STRING, Machine Learning, and WGCNA, to identify and analyze relevant targets, followed by Cytoscape analysis, DAVID‐based GO/KEGG enrichment, and AutoDock Vina molecular docking. This approach systematically elucidated key mechanisms by which food contaminants contribute to carcinogenesis across cancer types, with a focus on BRCA, PRAD, and COAD.

Since 1970, a wide range of environmental toxins, such as chemicals in tobacco smoke, air pollutants, and contaminants in food have been identified (Murrison, Brandt, Myers, and Hershey 2019). These toxins are prevalent in daily life, largely due to industrial growth, poses significant health risks by affecting both the respiratory and cardiovascular systems, with substantial evidence linking them to the development of various chronic diseases and cancers (Orru et al. 2017). For this study, we selected nine common dietary contaminants (glyphosate, PFOS, nitrosamines, PBDEs, methylmercury, dioxins, acrylamide, pyrrolizidine alkaloids, and aflatoxin) based on data from the National Institute of Environmental Health Sciences (NIEHS) and the Environmental Protection Agency (EPA). Our comprehensive analysis identified 69 targets intersecting with pan‐cancer pathways, which were further refined through PPI analysis and GO/KEGG enrichment analyses. We focused specifically on BRCA, PRAD, and COAD cancers to elucidate potential mechanisms linking these contaminants to carcinogenesis.

In BRCA, our analysis identified 49 hub targets, with JUN emerging as a pivotal regulator. Notably, the PI3K‐Akt pathway emerged as particularly significant, given its well‐established role in breast cancer development and progression (Browne et al. 2024). This finding aligns with prior research, particularly the study by Kavarthapu R et al., which demonstrated that JUN, a key target identified in our study, serves as a crucial regulator of the PI3K‐Akt pathway and significantly contributes to breast cancer progression (Kavarthapu, Anbazhagan, and Dufau 2021). Similarly, EGFR, another key target identified in our study by machine learning algorithms and WGCNA, has been shown to drive tumor growth and metastasis in BRCA (Sigismund, Avanzato, and Lanzetti 2018). RNA sequencing from TCGA data revealed significantly lower JUN expression in tumors compared to normal tissues. While higher JUN expression correlated with improved survival outcomes in BRCA patients, we did not observe significant associations between EGFR expression levels and patient survival. GSEA further indicated JUN's association with hypoxia, EMT, oxidative phosphorylation, and E2F targets, which is in line with previous research on BRCA (Xiong et al. 2022; R. Zeng, Peng, and Peng 2022; Zhou et al. 2024). Additionally, molecular docking analysis demonstrated stable binding affinities between the contaminants and JUN, suggesting direct interactions that may contribute to carcinogenesis. These results not only corroborate prior research but also provide novel insights into the molecular mechanisms underlying contaminant‐induced BRCA, highlighting JUN as potential therapeutic targets.

For PRAD, 44 hub targets were identified, with MAPK14 as a core driver. Pathway analysis highlighted the PI3K‐Akt pathway, implicated in prostate cancer through its regulation of cell proliferation and apoptosis (Hashemi et al. 2023; Shorning et al. 2020). TCGA data showed reduced MAPK14 expression in tumors, yet lower levels correlated with longer survival, a paradox emphasizing its context‐dependent duality. We hypothesize that early tumorigenesis involves MAPK14 loss to evade apoptosis, while advanced tumors exploit its elevation for invasion and therapy resistance, as observed in castration‐resistant disease (Cheung et al. 2021). Consequently, high intratumoral MAPK14 levels correlate with aggressive phenotypes and poorer survival. This dual role tumor suppressor in early stages and potential oncogenic driver in advanced stages is a recognized feature of several critical signaling pathways and highlights the complexity of targeting MAPK14 for therapeutic purposes. GSEA linked MAPK14 to EMT, inflammatory response, and DNA repair. This finding is consistent with previous research on PRAD (Armstrong et al. 2023; Teng et al. 2021). Molecular docking analysis revealed stable binding affinities between food contaminants and MAPK14, indicating direct interactions that may drive carcinogenesis. This finding extends current research by establishing a molecular connection between dietary exposures and PRAD, highlighting MAPK14 as a functional nexus for contaminant effects.

Furthermore, our analysis identified 48 core targets associated with food contaminant‐induced COAD, with CDC42 emerging as a particularly significant candidate. CDC42 displayed significantly reduced expression in tumor compared to normal samples, and lower CDC42 levels correlated with poorer survival outcomes, consistent with recent findings linking CDC42 dysregulation to aggressive cancer phenotypes (Justilien et al. 2019). GSEA indicated that in CDC42, the signaling pathways associated with EMT, inflammatory response, and interferon gamma response exhibited a positive correlation, whereas those related to myc targets had a negative correlation. This discovery aligns with previous studies on COAD (Chang et al. 2024; Xin et al. 2022). Molecular docking analysis revealed stable binding affinities between food contaminants and CDC42, pointing to direct interactions that may drive carcinogenesis. These findings not only corroborate existing research but also highlight CDC42 as both a potential therapeutic target and biomarker for contaminant‐associated COAD

Our study employs an innovative approach intergrating PPI analysis, machine learning algorithms, WGCNA, and molecular docking simulations to comprehensively explore the molecular mechanisms of carcinogenesis induced by nine food contaminants. This integrated methodology not only pinpoints crucial targets and pathways linking food contaminants to cancer development but also uncovers the complex interplay between environmental factors and cellular processes. A key finding was that a single contaminant can impact different cancers via distinct targets; for example, PFOS appears to target JUN in BRCA but MAPK14 in PRAD. Beyond these specific mechanisms, our pan‐contaminant design uncovered shared molecular targets (e.g., JUN, MAPK14, CDC42) and convergent pathways, such as PI3K‐Akt signaling across these diverse contaminants. Such commonality implies that real‐world coexposure to several contaminants may lead to synergistic effects on oncogenic processes, particularly when different contaminants act on the same target, as suggested by our docking results for MAPK14.

Compared to earlier studies that often focused on single compounds or employed narrower methodological scopes (Cao et al. 2022; H. Li et al. 2025; Lu et al. 2022), our work provides a more comprehensive and systematic evaluation of contaminant‐induced carcinogenesis. By integrating multi‐database screening with advanced computational techniques, we offer a broader perspective on how the exposome influences cancer‐related pathways. This strategy has enabled the identification of central regulatory nodes that could inform the development of preventive or therapeutic strategies effective against multiple contaminants.

Our molecular docking results indicate that food contaminants bind to functionally critical residues of JUN, MAPK14, and CDC42, suggesting specific mechanistic interference with their biological roles. For JUN, contaminants interact with residues within the transactivation and basic domains of the bZIP motif (e.g., Arg‐16, Gln‐12, Arg‐21), potentially impairing the formation of the activator protein‐1 (AP‐1) complex and dampening inflammatory signaling. In MAPK14, the contaminant binds at the ATP pocket, engaging the hinge region (His‐107–Met‐109) and catalytic Asp‐150, mirroring the binding mode of the established inhibitor SB203580. This suggests competitive inhibition of ATP binding, likely suppressing phosphotransferase activity and disrupting downstream MAPK signaling involved in inflammation and stress response. For CDC42, the contaminant targets Switch‐I (Gly‐15, Lys‐16) and Switch‐II (Asp‐57, Thr‐58) regions, along with Arg‐186 in the polybasic tail, key elements for GTP hydrolysis and effector binding. This interaction is predicted to lock CDC42 in an inactive, GDP‐bound state, impairing PAK1 binding and providing a structural basis for disrupted actin dynamics and epithelial integrity. Collectively, these findings provide mechanistic hypotheses for contaminant‐induced dysregulation of inflammation, stress response, and cytoskeletal organization, supporting further experimental validation of these target–contaminant interactions in carcinogenesis.

Notably, we found that pivotal regulators such as JUN and CDC42 display altered expression in tumors and are correlated with patient survival. These findings offer a foundation for developing targeted inhibitors that could simultaneously mitigate the effects of multiple contaminants by blocking common targets. In addition, our results may support public health initiatives aimed at reducing exposure to high‐risk contaminants and inform the development of biomarkers for early cancer detection in high‐risk populations. While further experimental validation is necessary to fully elucidate the effects of contaminant mixtures, this study establishes a foundational framework for understanding how environmental stressors collectively promote cancer through shared molecular mechanisms.

Despite the comprehensive nature of our study, several limitations should be acknowledged. First, our analysis relies heavily on in silico methods, and there is an inability to directly extrapolate the in vitro docking results to in vivo effects. The identified targets and pathways need to be validated through experimental studies, including using animal models, which were lacking in the current study. Additionally, metabolite toxicity was not considered in our research, which could impact the overall conclusions. Moreover, the potential synergistic effects of multiple contaminants, which are often present in real‐world scenarios, were not fully explored in this study. Future research should investigate the combined effects of contaminants on carcinogenesis, as this could provide more accurate insights into the risks posed by contaminated food.

## 5 Conclusion

In conclusion, our study offers groundbreaking insights into how food contaminants contribute to cancer development by uncovering critical molecular targets and pathways, specifically identifying JUN, CDC42, and MAPK14 as potential molecular targets associated with contaminant‐induced carcinogenesis. While acknowledging certain limitations, this research establishes a foundation for future investigations to validate these discoveries. Although these findings deepen our understanding of the molecular mechanisms behind contaminant‐driven cancer, it should be noted that at present, the feasibility of translating these targets into practical applications in cancer prevention and treatment, such as the development of target drugs, has not been fully explored. Further research is needed to explore their potential in formulating more effective global strategies to alleviate the cancer burden.

## Author Contributions

**Yi Gu**: conceptualization, methodology, software. **Wenzhu Lou**: methodology, validation, visualization, writing – original draft. **Shuaishuai Huang**: writing – review and editing, formal analysis, resources, supervision. **Feiyan Mao**: validation, formal analysis, supervision, resources. **Lian Tan**: validation, software, formal analysis, data curation. **Zhiyan Wang**: project administration, conceptualization, resources, supervision. **Bangsheng Chen**: conceptualization, project administration, funding acquisition. **Maomao Li**: conceptualization, investigation.

## Funding

This research was supported by The Natural Science Foundation of Ningbo (2022J039), Zhejiang Province Traditional Chinese Medicine Science and Technology Project (2026ZL0797), and The Health Industry Science and Technology Plan Project of Zhejiang Province (2026778552).

## Conflicts of Interest

The authors declare no conflicts of interest.

## Supporting information




**Fig. S1. Key WGCNA Modules Identified in TCGA‐BRCA Dataset**. (A) Sample clustering dendrograms with leaves corresponding to each sample. (B) Determination of the soft‐thresholding power. (C) Dendrogram of differentially expressed genes clustered based on a dissimilarity measure (1‐TOM). (D) Module‐trait relationships were established to assess correlations with tumor occurrence. (E) The heatmap of Eigengene adjacency.

**Fig. S2. Key WGCNA Modules Identified in TCGA‐PRAD Dataset**. (A) Sample clustering dendrograms with leaves corresponding to each sample. (B) Determination of the soft‐thresholding power. (C) Dendrogram of differentially expressed genes clustered based on a dissimilarity measure. (D) Module‐trait relationships were established to assess correlations with tumor occurrence. (E) The heatmap of Eigengene adjacency.

**Fig. S3. Key WGCNA Modules Identified in TCGA‐COAD Dataset**. (A) Sample clustering dendrograms with leaves corresponding to each sample. (B) Determination of the soft‐thresholding power. (C) Dendrogram of differentially expressed genes clustered based on a dissimilarity measure. (D) Module‐trait relationships were established to assess correlations with tumor occurrence. (E) The heatmap of Eigengene adjacency.

## Data Availability

The datasets used in this study are all publicly available.
