# Supplementary figures and images for: Unraveling the Carcinogenic Mechanisms of Food Contaminants: An Integrated in Silico Framework Combining Network Toxicology, Machine Learning, and Molecular Docking
Source: J Food Sci. 2025 Nov 17;90(11):e70697. doi: 10.1111/1750-3841.70697 (PMC12621292; doi:10.1111/1750-3841.70697)

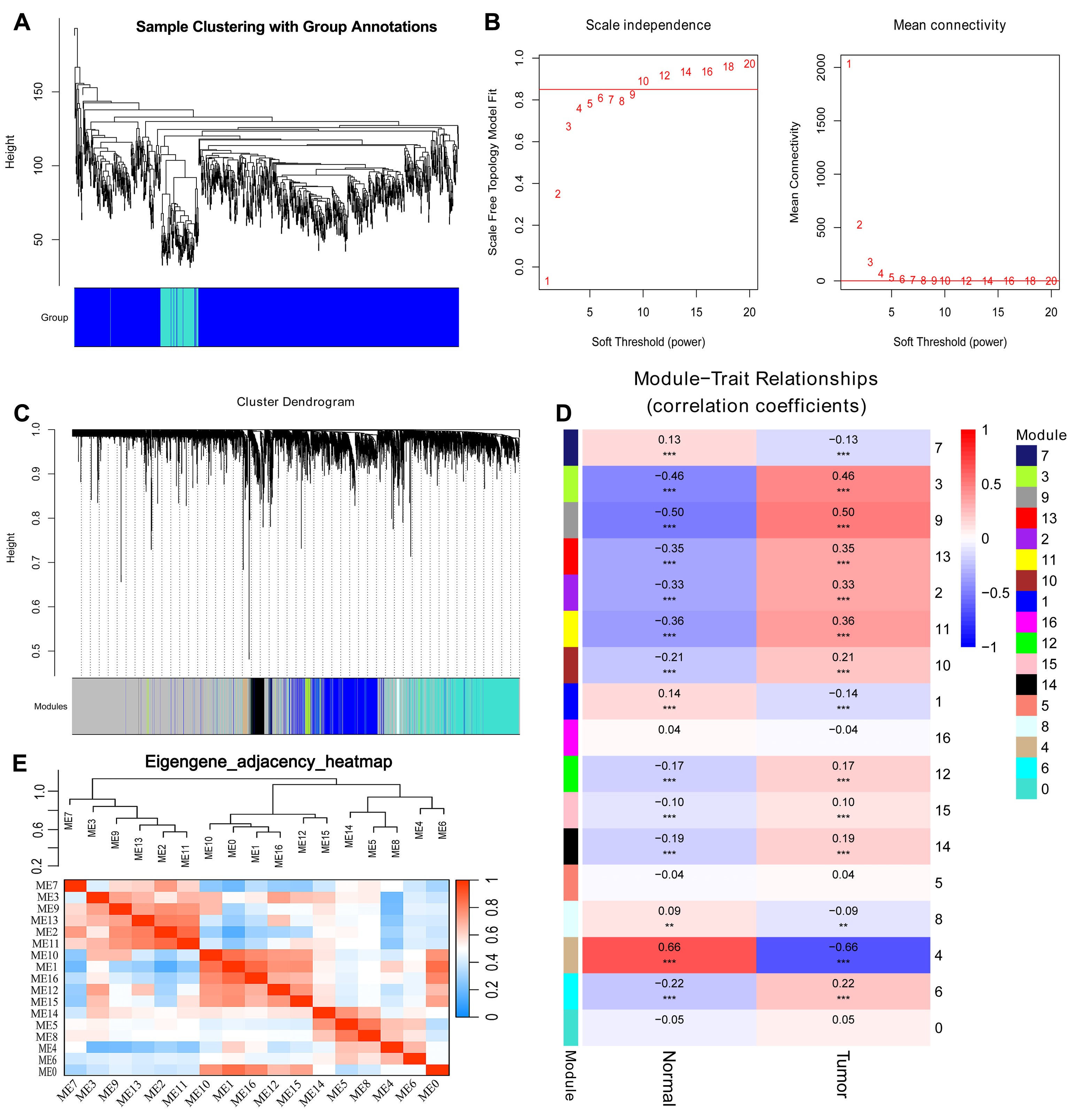

Supplement: Supplementary file 1 — Fig. S1. Key WGCNA Modules Identified in TCGA‐BRCA Dataset. (A) Sample clustering dendrograms with leaves corresponding to each sample. (B) Determination of the soft‐thresholding power. (C) Dendrogram of differentially expressed genes clustered based on a dissimilarity measure (1‐TOM). (D) Module‐trait relationships were established to assess correlations with tumor occurrence. (E) The heatmap of Eigengene adjacency. [file JFDS-90-0-s002.jpg]

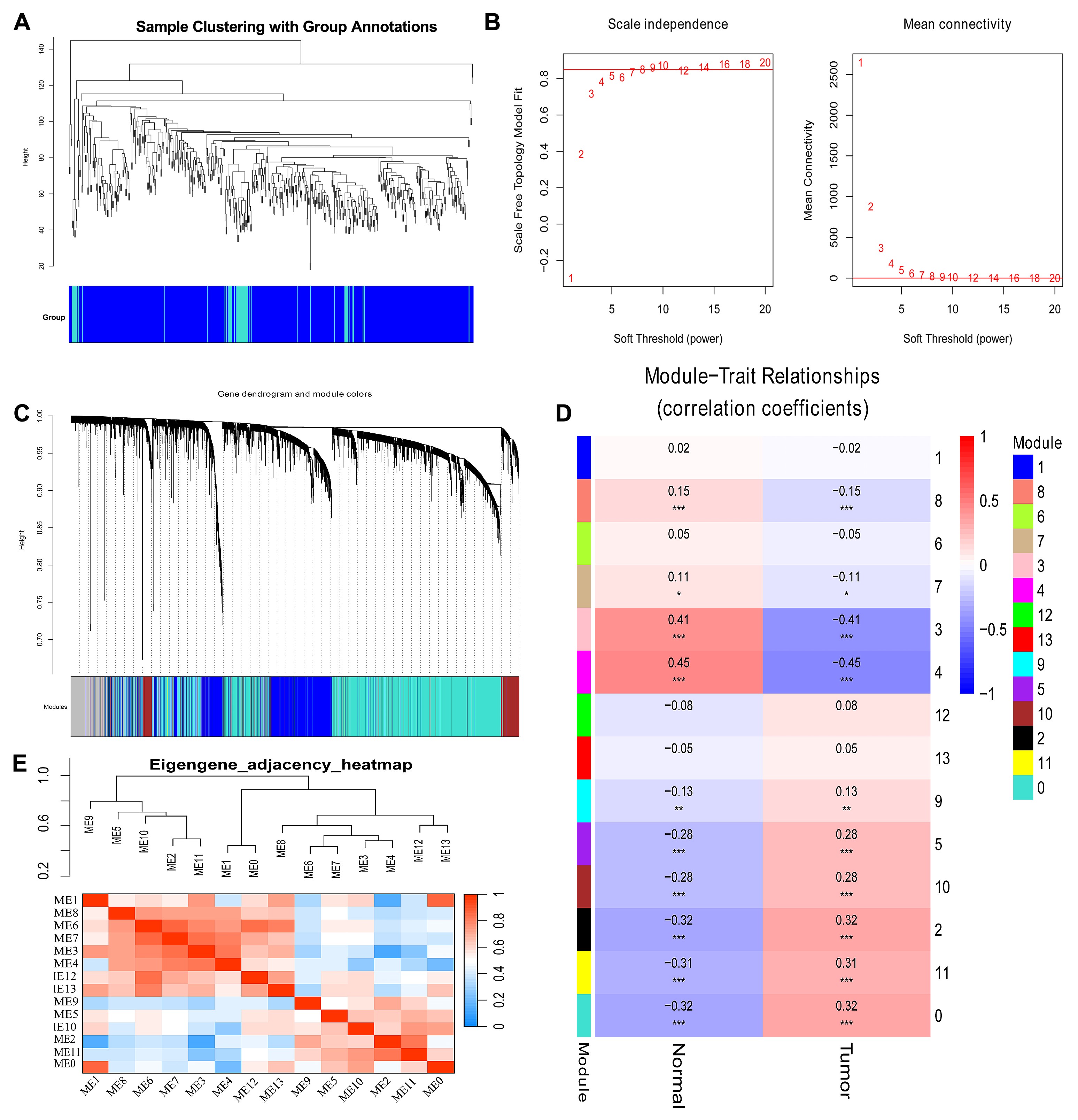

Supplement: Supplementary file 2 — Fig. S2. Key WGCNA Modules Identified in TCGA‐PRAD Dataset. (A) Sample clustering dendrograms with leaves corresponding to each sample. (B) Determination of the soft‐thresholding power. (C) Dendrogram of differentially expressed genes clustered based on a dissimilarity measure. (D) Module‐trait relationships were established to assess correlations with tumor occurrence. (E) The heatmap of Eigengene adjacency. [file JFDS-90-0-s003.jpg]

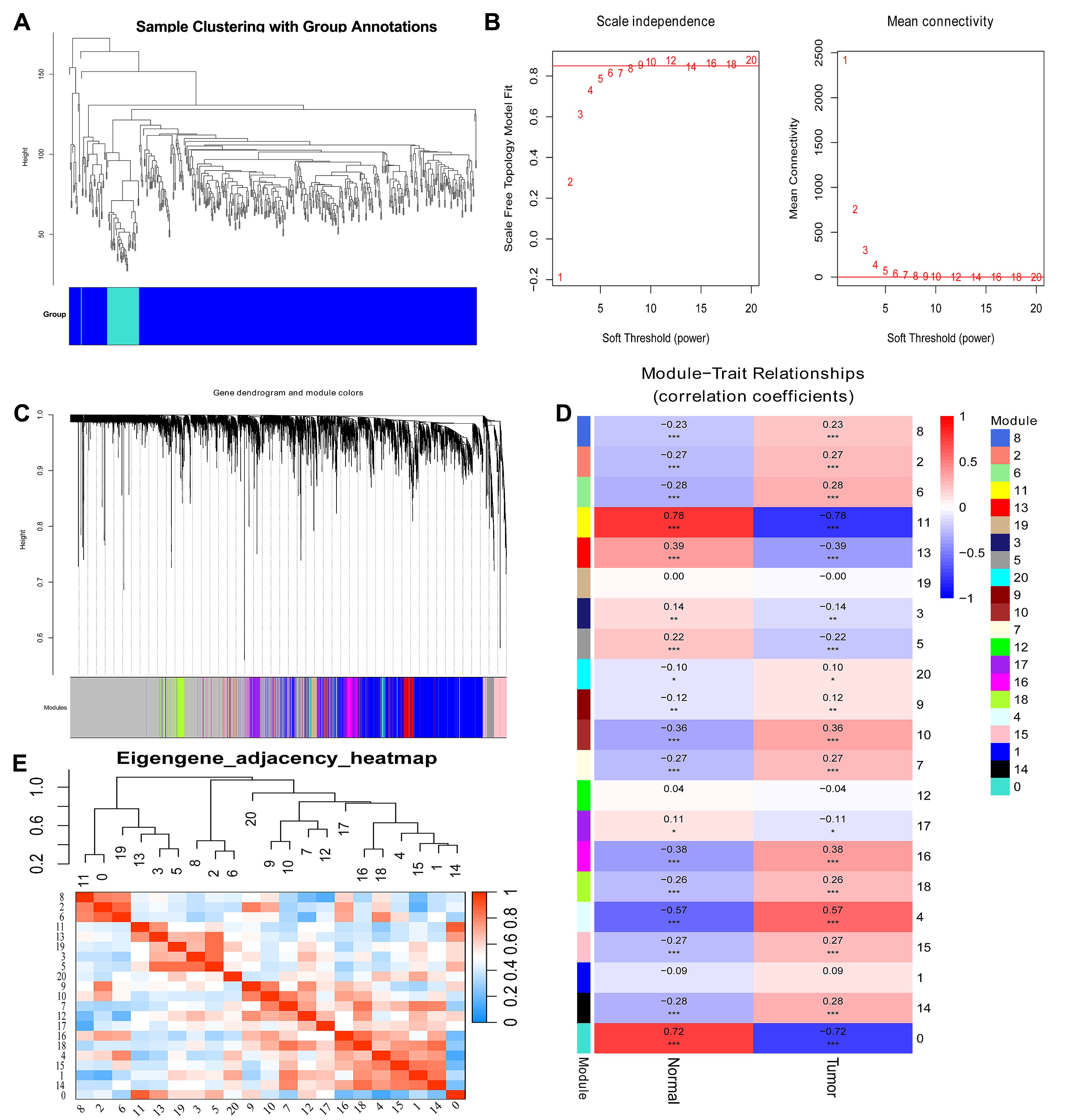

Supplement: Supplementary file 3 — Fig. S3. Key WGCNA Modules Identified in TCGA‐COAD Dataset. (A) Sample clustering dendrograms with leaves corresponding to each sample. (B) Determination of the soft‐thresholding power. (C) Dendrogram of differentially expressed genes clustered based on a dissimilarity measure. (D) Module‐trait relationships were established to assess correlations with tumor occurrence. (E) The heatmap of Eigengene adjacency. [file JFDS-90-0-s001.jpg]
